# Use of the “Ru‐^1^O_2_‐Hydrazide” System Catalyzed by Metallic Ruthenium Complexes to Decipher the Interaction Between Microbes and Host Cancer Cells

**DOI:** 10.1002/advs.76240

**Published:** 2026-06-22

**Authors:** Amin Sun, Kaihong Wang, Haifu Sun, Kemei Tao, Xiang Li, Xiuhua Zhao, Shuang Qiu

**Affiliations:** ^1^ Key Laboratory of Forest Plant Ecology Ministry of Education Northeast Forestry University Harbin Heilongjiang China; ^2^ College of Food and Health Northeast Forestry University Harbin Heilongjiang China; ^3^ Hubei Province Key Laboratory of Biotechnology of Chinese Traditional Medicine College of Health Science and Engineering Hubei University Wuhan China; ^4^ Shenzhen GeneSeqTools Bio‐Science & Technology Co., Ltd Shenzhen China

**Keywords:** photocatalysis, proximity labeling, bacteria‐host cancer cell interactions, mechanism of interaction, drug screening

## Abstract

The dynamic interplay between bacteria and host cancer cells plays a critical role in tumor microenvironment modulation, bacterial pathogenesis, and potential oncotherapy applications. However, traditional methods often fail to capture transient or spatially restricted molecular interactions at the bacteria‐cancer cell interface. Proximity labeling has emerged as a promising technology for capturing the interaction between bacteria and host‐cancer cell. Photocatalytic proximity labeling is more efficient, faster, and higher in resolution than enzyme‐catalyzed proximity labeling. Here, we employ photoactivatable proximity labeling technology—the “Ru‐^1^O_2_‐hydrazide” system to rapidly capture the interaction between bacteria and host cancer cells. This system is using the singlet oxygen (^1^O_2_) mechanism with biotin hydrazide by anchoring the photosensitizer Ru(bpy)_3_^2+^ on the bacteria surface to efficiently and discriminatively capture the bacteria‐host cancer cell interactions (BHIs). Furthermore, we established a quantitative strategy based on the “Ru‐^1^O_2_‐hydrazide” system to characterize bacteria‐host cell interaction strength. This strategy allows for systematic evaluation of drug effects on BHIs, offering novel insight into pharmaceutical modulation of bacteria‐host cancer cell crosstalk.

## Abstract 2

A novel photocatalytic proximity labeling system rapidly captures bacteria‐cancer interactions. Using a ruthenium‐catalyzed proximity labeling technique, this platform enables quantitative analysis and drug evaluation, offering new insights into bacterial modulation of tumors.

## 1 Introduction

The interactions between bacteria and host cell are widespread in different tissues and organs, playing a crucial role in maintaining physiological homeostasis and health, as well as driving disease development and therapy [1, 2, 3, 4, 5]. In order to understand how they regulate physiological states and unravel disease pathogenesis, extensive characterization of bacterial‐host cell interactions during specific physiological processes is essential [4]. Conventional biochemical approaches, such as in vitro culture models [6], microscopic imaging techniques [7], transcriptome or spatial transcriptome sequencing methods [8, 9, 10], and bioinformatics software prediction techniques [11], have all made significant contributions to the study of bacterial‐host cell interactions. However, these methods are limited in terms of timeliness, quantification, and selectivity, due to poor temporal resolution, the ease with which weak or transient interactions are lost, and the fact that they can only demonstrate the presence of interactions, rather than quantitatively studying bacteria‐host cell interactions. Consequently, as these methods can only demonstrate bacterial‐host cell interactions qualitatively, they are also unable to analyze the mechanism of these interactions precisely.

Proximity labeling is an emerging method widely used to capture cell‐to‐cell and biomolecular interactions. Proximity labeling strategies involve the use of an enzyme catalyst or small‐molecule photocatalyst that is directed to a cell–cell interaction environment through attachment to a surface protein or biomolecule of interest [12, 13, 14, 15]. Compared to enzyme‐catalyzed proximity labeling techniques, photocatalytic proximity labeling has the advantages of not requiring genetic engineering modifications and being unaffected by enzyme activity. The catalyst is then activated by applying an appropriate stimulus to induce tagging of neighboring substrates with a biotinylated probe. The biotinylated tags are subsequently enriched via streptavidin affinity purification and analyzed by methods such as mass spectrometry, gel imaging, microscopy, flow cytometry, or gene sequencing methods. Existing photocatalytic proximity labeling techniques include the µmap strategy (using iridium catalysts) [16, 17], the PhoXCELL technique (based on dibromofluorescein) [18], the Pho Tag technique (employing riboflavin) [19], and the “Ru‐^1^O_2_‐hydrazide” system (with ruthenium complexes) [20]. It also includes the recently reported “CINTER‐seq” technique [21] with pyropheophorbide‐a (PPa) and PATCH platform [22] with porphyrin‐based nanozymes in vivo for capturing cell‐cell interactions. These techniques are used to study cell‐cell interactions. The Sn^IV^ dihydroporphyrin e6 catalyst µmap‐red technique [23], metal‐iridium catalyst‐based, CAT‐Prox [24], PhotoCAX [25], CAT‐Ex [26], CAT‐S [27], CAT‐seq [28], dihydroporphyrin e6 catalyst‐based CAT‐Tissue [29], metal‐osmium based catalyzed bioorthogonal reactions, CAR‐NIR [30], BREF‐ID [31] and SeeID [32] platform can be used to study biomolecular interactions. These methods have provided a comprehensive set of tools for studying cell‐cell or biomolecule interactions, such as protein–protein and protein‐RNA interactions. However, they have not been utilized to study microbe‐host cell interactions. This gap highlights a promising opportunity, offering a powerful potential tool for investigating these critical interfaces.

Photocatalytic proximity labeling techniques are classified into three main types based on labeling mechanisms: singlet oxygen (^1^O_2_), hydroxyl radical, and carbene intermediate systems. Hydroxyl radicals last up to 100 µs, achieving a labeling radius of tens of nanometers. Singlet oxygen has a shorter lifetime than radicals, but can achieve a labeling radius of over ten nanometers. In contrast, carbene intermediates exhibit an extremely short half‐life (∼1 ns) and a limited labeling radius of 2 nm, making them suitable for detailed studies of biomacromolecule interactions such as those between proteins or nucleic acids. Both singlet oxygen and hydroxyl radicals offer broader labeling ranges, though hydroxyl radicals diffuse further and tend to introduce stronger background in applications like capturing cell–cell or microbe‐host interactions [13]. Therefore, singlet oxygen‐based labeling is preferable for better background control. Previous studies have reported that Ru(bpy)_3_^2+^ exhibits superior singlet oxygen generation capacity compared to dibromofluorescein, riboflavin, and other common photosensitizers. The Ru(bpy)_3_^2+^‐catalyzed proximity labeling method (“Ru‐^1^O_2_‐hydrazide” system [20]) exploits this mechanism by anchoring the photosensitizer to the cell surface, enabling efficient and highly discriminative capture of cell‐cell interactions (CCIs).

**SCHEME 1:**
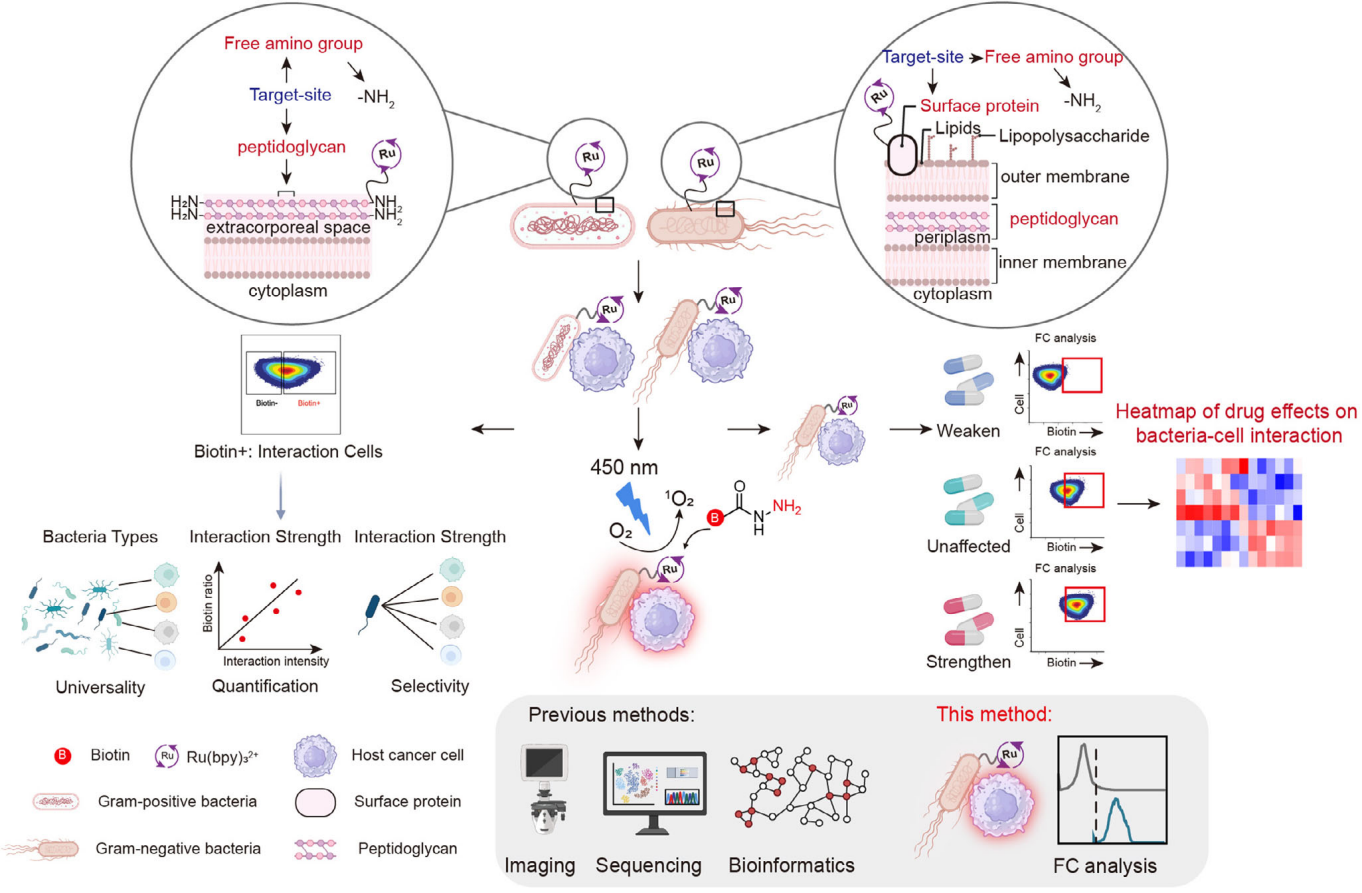
Photocatalytic proximity labeling of BHIs using the “Ru‐^1^O_2_‐hydrazide” system.

Herein, we use the “Ru‐^1^O_2_‐hydrazide” system as a new strategy to decipher the interaction between bacteria and host cancer cells. The photosensitive metal catalyst Ru(bpy)_3_^2^^+^ was loaded onto the free amino groups on the surfaces of Gram‐positive and Gram‐negative bacteria using activated, ester‐modified Ru(bpy)_3_^2+^. The Ru(bpy)_3_^2+^‐catalyzed proximity labeling reaction was triggered by blue LEDs (λmax = 450 nm) in the presence of biotin hydrazide, which was used as a substrate to label cells that interacted with the bacteria. This biotin signaling method enables the efficient and rapid capture of bacterial‐host cancer cell interactions (Scheme 1).

## 2 Results

### 2.1 Efficient Loading of Ru(Bpy)_3_^2+^ on Bacterial Surfaces and Its Catalytic Activity

**FIGURE 1:**
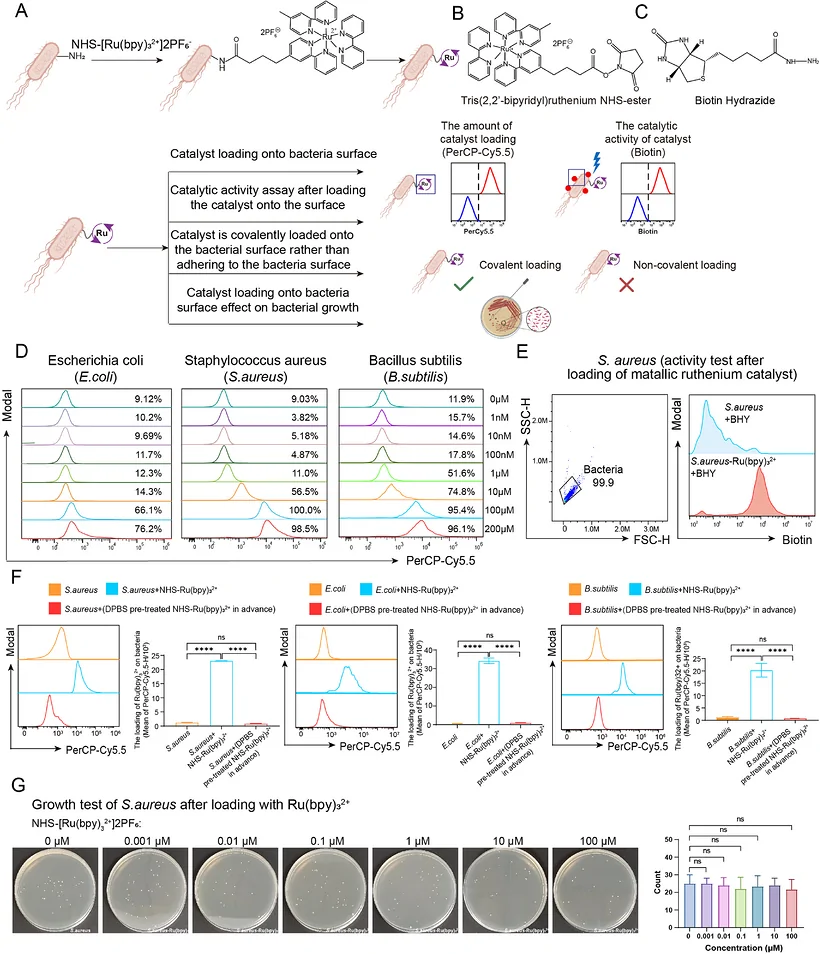
Loading of the Ru(bpy)_3_^2+^ onto the bacteria. (A) The schematic representation of Ru(bpy)_3_^2+^ loading onto the bacteria surface, its catalytic activity, and its effect on bacterial growth. (B, C) Chemical structure of Tris(2,2’‐bipyridyl)ruthenium NHS‐ester (NHS‐[Ru(bpy)_3_^2+^]2PF_6_^−^) and biotin hydrazide. (D) Anchoring Ru(bpy)_3_^2+^ onto *E. coli*, *S. aureus*, and *B. subtilis* by incubating with different concentration of NHS‐[Ru(bpy)_3_^2+^]2PF_6_^−^ at room temperature for 15 min. (E) Flow cytometry analysis of self‐biotinylation on *S. aureus* by bacteria surface‐anchored Ru(bpy)_3_^2+^. *S. aureus* was incubated NHS‐[Ru(bpy)_3_^2+^]2PF_6_^−^ (100 µm) to install Ru(bpy)_3_^2+^ on *S. aureus* surface, followed by washing three times with DPBS and treating with 100 µm biotin hydrazide then photo‐irradiated by 450 nm for 5 min. (F) Flow cytometry analysis and summary statistics showing the PerCP‐Cy5.5 signal, representing the amount of Ru(bpy)_3_^2+^ on *S. aureus* after incubation with NHS‐[Ru(bpy)_3_^2+^]2PF_6_^−^ or with DPBS pre‐treated NHS‐[Ru(bpy)_3_^2+^]2PF_6_^−^ (in which the NHS ester was hydrolyzed and inactivated). n = 3. (G) Bacterial growth plates and summary statistics showing bacterial growth 24 h after incubating the bacteria with different concentration of Ru(bpy)_3_^2+^. n = 3. (n: number of biological replicates. ns *p*>0.05; ^****^ *p*<0.0001.).

Metabolic labeling can also load bioorthogonal groups such as TCO onto the surface of bacteria, and Ru(bpy)_3_^2+^ can be loaded via Ru(bpy)_3_^2+^‐tetrazine (Tz). However, metabolic labeling requires the bacteria to be exposed to a large amount of labeled medium, which affects their metabolic states and thus their ability to interact with host cell [33, 34, 35, 36]. This method is also not suitable for modifying bacteria in order to study their interaction with host cancer cells. Therefore, we need to design a method that can load Ru(bpy)_3_^2+^ onto bacterial surfaces efficiently and universally. The cell wall compositions of Gram‐positive and Gram‐negative bacteria differ significantly. Gram‐positive bacteria have a simple cell wall consisting mainly of peptidoglycan, which consists of polysaccharide chains and cross‐linked polypeptide chains [37]. The cell wall of Gram‐negative bacteria is complex, consisting of an inner peptidoglycan layer and an outer layer of lipids, lipopolysaccharides, and proteins [38, 39]. We selected the free amino groups on the peptidoglycan in Gram‐negative bacteria and the amino groups on surface proteins in Gram‐positive bacteria as targets for Ru(bpy)_3_^2+^ loading. Ru(bpy)_3_^2+^ was then loaded onto bacterial surfaces via an activated ester reaction with amino groups using NHS‐[Ru(bpy)_3_^2+^]2PF_6_^−^ (Figure 1A). This approach was successfully applied to *Escherichia coli* *(E. coli)*, *Staphylococcus aureus* *(S. aureus)*, and *Bacillus subtilis* *(B. subtilis)*. As reported in the literature, photo‐excited *Ru(bpy)_3_^2+^ undergoes fluorescence emission at *λ*_max_ = 615 nm with inherent intensity. Thus, the success of Ru(bpy)_3_^2+^ attachment on bacteria surface could be characterized by flow cytometry using the channel of PerCP‐Cy5.5 (695/40 nm). Flow cytometry analysis that 100 µm NHS‐[Ru(bpy)_3_^2+^]2PF_6_^−^ was sufficient to achieve saturated Ru(bpy)_3_^2+^ loading on the surfaces of three bacterial strains (Figure 1B–D). The ability of Ru(bpy)_3_^2+^ to catalyze bacterial self‐labeling was examined using Ru(bpy)_3_^2+^‐loaded *S. aureus*. The addition of the biotin hydrazide substrate and blue light irradiation at 450 nm for 5 min resulted in the surface of the bacteria being labeled with a strong biotin signal by flow cytometry analysis. This suggests that Ru(bpy)_3_^2+^ is able to maintain its catalytic activity after being loaded onto the bacterial surface (Figure 1E and Figure S1). We then designed experiments to verify that the Ru(bpy)_3_^2+^ was covalently attached to amino groups on bacterial surfaces rather than simply adhering to them. NHS‐[Ru(bpy)_3_^2+^]2PF_6_^−^ was inactivated by DPBS treatment before being incubated with bacteria. The loading of Ru(bpy)_3_^2+^ onto the *E. coli*, *S. aureus*, and *B. subtilis* were then detected by flow cytometry. It was found that the PerCP‐Cy5.5 signals were the same as those of the bacteria in the untreated group (Figure 1F and Figure S2). This indicated that Ru(bpy)_3_^2+^ was covalently attached to the amines of the three bacteria rather than adhering to their surfaces. Additionally, we explored the effect of Ru(bpy)_3_^2+^ loading on bacterial growth and found it to be essentially unaffected (Figure 1G and Figure S3). Therefore, Ru(bpy)_3_^2+^ can be used as a photocatalytic proximity labeling tool to capture the interaction between bacteria and host cancer cells.

### 2.2 Detection and Quantification of the Interaction Intensity Between *S. aureus* and HeLa Cells

**FIGURE 2:**
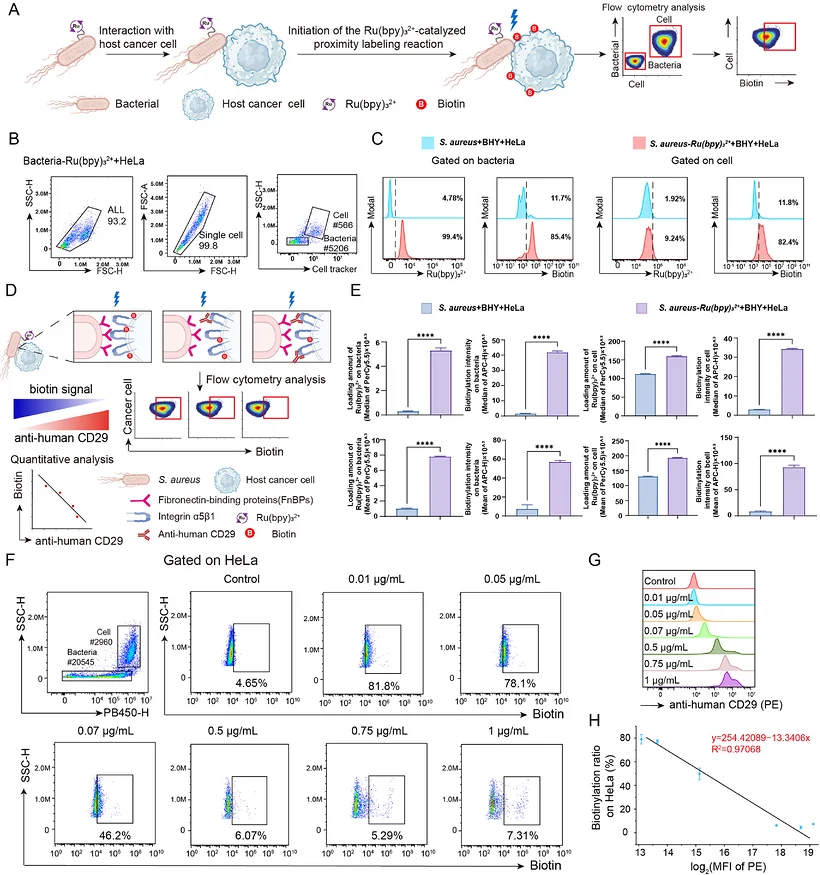
Use of the “Ru‐^1^O_2_‐hydrazide” system for quantitatively study the interaction between bacteria and host cancer cell. (A) Schematic representation of the bacteria‐host cell proximity labeling catalyzed by Ru(bpy)_3_^2+^ in BHIs. (B, C, E) Flow cytometry histogram analysis and summary statistics showing the median and mean fluorescence intensity of Ru(bpy)_3_^2+^ and biotinylation of *S. aureus* and HeLa after co‐culture of HeLa with either *S. aureus* or *S. aureus*‐*Ru(bpy)_3_^2+^* interact with HeLa. The background is defined as the signal produced on HeLa when incubate with *S. aureus* without surface‐anchored Ru(bpy)_3_^2+^. Incubation time, 2 h; irradiation time, 5 min. n = 3. (D) Schematic illustration of the quantitative study of the interaction between *S. aureus* and HeLa. (F) Flow cytometry analysis of the biotinylation ratio on HeLa cells pretreated with anti‐human CD29 antibody at concentrations ranging from 0.01 to 1 µg/mL (0.01, 0.05, 0.07, 0.5, 0.75, and 1 µg/mL), followed by incubation with *S. aureus‐Ru(bpy)_3_^2+^* for 2 h. The background is defined as the signal produced on HeLa when incubated with *S. aureus* without surface‐anchored Ru(bpy)_3_^2+^. Incubation time, 2 h; irradiation time, 5 min. n = 3. (G) Flow cytometry histogram analysis of the blocking effects of different concentrations of anti‐human CD29 antibody on HeLa cells. Correlation analysis between biotinylation ratio on HeLa cells (pretreated with different concentrations of anti‐human CD29) and the median fluorescence intensity (MFI) of PE‐anti human CD29 following incubation with *S. aureus*‐*Ru(bpy)_3_^2+^*. (H) Correlation analysis between biotinylation ratio on HeLa cells (pretreated with different concentrations of anti‐human CD29) and the MFI of PE‐anti human CD29 following incubation with *S. aureus*‐*Ru(bpy)_3_^2+^*. n = 3. (n: number of biological replicates. ^****^ *p*<0.0001.).

Exploring whether the “Ru‐^1^O_2_‐hydrazide” system can be used to capture interactions between bacteria and host cancer cells is a prerequisite for studying these interactions quantitatively. So far, no researchers have used photocatalytic proximity labeling to study these interactions. In order to investigate the potential of the “Ru‐^1^O_2_‐hydrazide” system for capturing and analyzing these interactions, we selected a model representing *S. aureus* infection of epithelial cells, a key process in *S. aureus* pathogenesis. Cervical cancer cell (HeLa) is a type of cancerous epithelial cell, and an *S. aureus* infection model using HeLa was employed to explore the ability of the “Ru‐^1^O_2_‐hydrazide” system to capture BHIs (Figure 2A). First, NHS‐[Ru(bpy)_3_^2+^]2PF_6_^−^ was loaded onto *S. aureus* (*S. aureus‐Ru(bpy)_3_^2+^*), *S. aureus* and *S. aureus‐Ru(bpy)_3_^2+^* were co‐culture with HeLa cells respectively, and then trigger the Ru(bpy)_3_^2+^ mediated labeling reaction. In the HeLa interaction group with Ru(bpy)_3_^2+^ loaded *S. aureus*, we observed that 99.4% of *S. aureus* were PerCP‐Cy5.5 positive and 85.4% were biotin‐positive, whereas 9.24% of HeLa cells were PerCP‐Cy5.5 positive and 82.4% were biotin‐positive (Figure 2B,C,E). This suggests that Ru(bpy)_3_^2+^ loading onto *S. aureus* does not transfer to HeLa cells during interaction and that the labeling can be used to capture bacteria‐host cancer cell interactions via the labeled bacteria. To confirm the labeling mechanism, GSH quenching experiments demonstrated that biotin labeling is mediated by singlet oxygen (Figure S4). Furthermore, bacteria–cell contact labeling assays performed at varying densities, combined with high‐content imaging, revealed that labeling depends on direct interactions between adjacent bacteria and host cancer cells (Figure S5).

Quantitatively distinguishing the strength of interactions is crucial for a deep molecular‐level understanding of the interplay between bacteria and host cancer cells. To investigate whether the “Ru‐^1^O_2_‐hydrazide” system is suitable for quantitative analysis of these interactions, we constructed an antibody‐blocking model system targeting the interaction between *S. aureus* and HeLa cells. It has been reported that the interaction between *S. aureus* and host cancer cell is mediated by fibronectin‐binding proteins on the bacterial surface and integrin α5β1 on the host cell surface. Integrin α5β1 is a key cell surface adhesion receptor primarily composed of two subunits: α5 (CD49e) and β1 (CD29). Within this complex, CD29 serves as the core functional subunit, playing a dominant role in mediating cell adhesion and signal transduction, while CD49e plays an auxiliary role, primarily responsible for recognizing fibronectin and assisting CD29 in specific ligand binding. The core function of α5β1 is to specifically recognize and bind to fibronectin in the extracellular matrix, thereby mediating the adhesion of *S. aureus* to host cells [40].

In this study, we first validated the responsiveness of the “Ru‐^1^O_2_‐hydrazide” system to the intensity of interactions mediated by the aforementioned specific molecular pair. HeLa cells were treated with different concentrations of anti‐human CD29 antibodies to achieve gradient regulation of CD29 expression levels on the cell surface.  *S. aureus*‐Ru(bpy)_3_^2+^ was then co‐incubated with six groups of HeLa cells exhibiting different surface CD29 expression levels for 2 h, triggering the Ru(bpy)_3_^2+^‐catalyzed proximity labeling reaction. Subsequently, the biotin labeling levels were detected on six groups of HeLa cells with different expression levels (Figure 2D,G). The experimental results showed that the biotin labeling signal obtained via the “Ru‐^1^O_2_‐hydrazide” on the surface of HeLa cells was positively correlated with the blockade degree of CD29 on the cell surface (i.e., negatively correlated with the working concentration of anti‐human CD29 antibodies), and a significant linear correlation was observed between the biotinylation signal and the CD29 blockade degree (Figure 2F,H).

Furthermore, we established two models of *S. aureus* adhesion to HeLa cells. In the first model, the *S. aureus*‐to‐HeLa ratio was fixed at 10:1, and the co‐incubation time was varied (1, 2, 4, and 6 h) to create a time‐dependent adhesion gradient. In the second model, the co‐incubation time was fixed, and the *S. aureus*‐to‐cell ratios were varied (10: 1, 5: 1, 1: 1, and 0.5: 1) to create a ratio‐dependent adhesion gradient, after which the Ru(bpy)_3_^2+^‐catalyzed proximity labeling reaction was triggered. Analysis of the results from both model systems revealed a strong linear correlation between the biotin labeling signals on HeLa cells and the bacterial adhesion levels under identical conditions (Figures S6 and S7). Collectively, these results demonstrate that the “Ru‐^1^O_2_–hydrazide” system enables quantitative study of bacteria and host cancer cell interactions, offering a valuable tool for capturing dynamic changes during infectious pathogenesis and for screening drug candidates that modulate these interactions.

### 2.3 Evaluating the Potential of the “Ru‐^1^O_2_‐Hydrazide” System to Selectively Capture the Strength of Bacterial‐Host Cancer Cell Interactions

**FIGURE 3:**
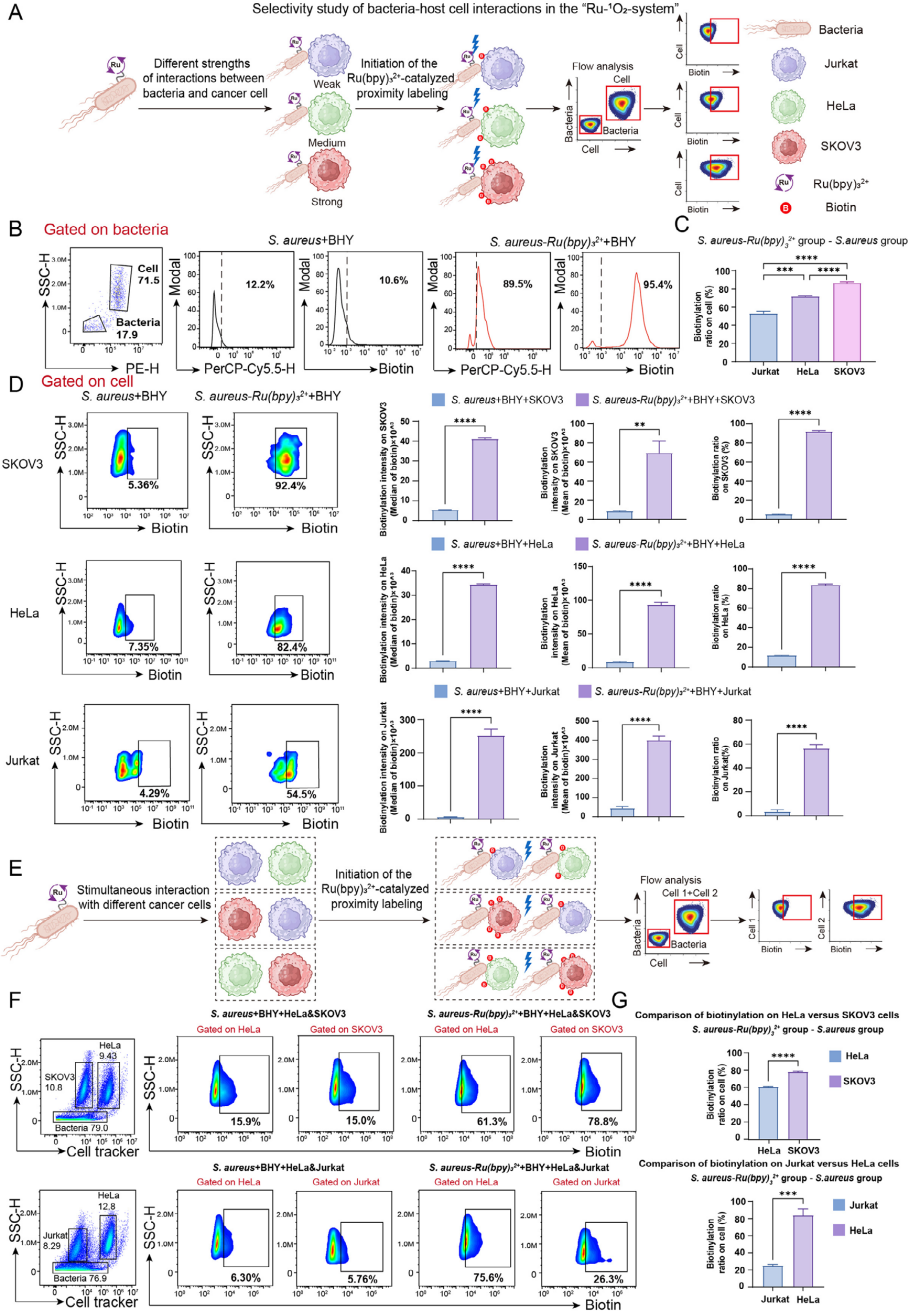
Use of the “Ru‐^1^O_2_‐hydrazide” system for selective study of the interaction between bacteria and different host cancer cells. (A) The schematic representation of Ru(bpy)_3_^2+^‐anchored *S. aureus* interacting with different host cancer cells, including Jurkat, HeLa, and SKOV3. (B) Flow cytometry analysis of the anchoring Ru(bpy)_3_^2+^ amount and biotinylation level of *S. aureus* when *S. aureus* or *S. aureus*‐*Ru(bpy)_3_^2+^* interacts with SKOV3 cells. (C) Summary statistics showing the net biotinylation ratio on Jurkat, HeLa, and SKOV3 cells. The net biotinylation ratio is calculated as the difference in biotinylation signal between the experiment group (incubated with *S. aureus*‐*Ru(bpy)_3_^2+^*) and the control group (incubated with *S. aureus*). The background is defined as the signal produced on Jurkat, HeLa, and SKOV3 after incubation with *S. aureus* without surface‐anchored Ru(bpy)_3_^2+^. (D) Flow cytometry analysis and summary statistics showing the ratio, median, and mean fluorescence intensity on Jurkat, HeLa, and SKOV3 cells after interaction with *S. aureus*‐*Ru(bpy)_3_^2+^*. The background is defined as the signal produced on Jurkat, HeLa, and SKOV3 after incubation with *S. aureus* without surface‐anchored Ru(bpy)_3_^2+^. Incubation time, 2 h; irradiation time,5 min. n = 3. (E) Schematic representation of Ru(bpy)_3_^2+^‐anchored *S. aureus* interacting simultaneously with two types of host cancer cells (e. g., Jurkat and HeLa, or HeLa and SKOV3). (F, G) Flow cytometry analysis and summary statistics showing the biotinylation ratio on Jurkat, HeLa, and SKOV3 after *S. aureus*‐*Ru(bpy)_3_^2+^* interacts with host cancer cells. The background is defined as the signal produced on Jurkat, HeLa, and SKOV3 after incubation with *S. aureus* without surface‐anchored Ru(bpy)_3_^2+^. Incubation time, 2 h; irradiation time, 5 min. n = 3. (n: number of biological replicates. ^**^ *p*<0.01; ^***^ *p*<0.001; ^****^ *p*<0.0001.).

To further demonstrate the selectivity of the “Ru‐^1^O_2_‐hydrazide” system for capturing interactions between bacteria and different host cancer cells, we used Ru(bpy)_3_^2+^‐loaded *S. aureus* as a probe to interact with three types of cells: two cancerous epithelial cell lines (SKOV3 & HeLa) and one non‐epithelial immune cell line (Jurkat). The biotinylation ratio on SKOV3, HeLa, and Jurkat were 92.4%, 82.4%, and 54.5% respectively (Figure 3A–D and Figures S8 and S9). The markedly higher biotinylation ratios on epithelial cancer cells compared to Jurkat cells aligns with the well‐documented susceptibility of epithelial cells to *S. aureus* infection [41, 42]. To investigate whether *S. aureus* interacts more strongly with SKOV3 than with HeLa cells, we designed the following experiments. First, we prepared two types of cell mixtures: SKOV3 mixed with HeLa (1: 1 ratio) and HeLa mixed with Jurkat (1: 1 ratio). These mixtures were then used in co‐culture with *S. aureus‐Ru(bpy)_3_^2+^*. 450 nm LED light triggered *Ru(bpy)_3_^2+^*‐catalyzed proximity labeling reactions, enabling detection of the biotinylation on each cell type in the different cell mixtures. The results indicated that the biotinylation ratios on SKOV3 and HeLa were 78.8% and 61.3% respectively, while those on HeLa and Jurkat were 75.6% and 26.3% respectively (Figure 3E–G and Figures S10 and S11). These results clearly indicate that *S. aureus* interacts more strongly with SKOV3 cells than with HeLa cells. Furthermore, *S. aureus* exhibits stronger interactions with both HeLa and SKOV3 cells than with Jurkat cells, consistent with its known epithelial cell tropism. This finding demonstrates that the “Ru‐^1^O_2_‐hydrazide” system can differentiate the interaction strengths of bacteria with different host cancer cells in mixed co‐cultures. This finding provides a method for identifying susceptible pathogens in complex cellular environments. To validate the generalizability of the “Ru‐^1^O_2_‐hydrazide” system for detecting bacteria‐host interactions, we applied *S. aureus‐Ru(bpy)_3_^2+^* to interact with murine primary splenocytes and triggered Ru(bpy)_3_^2+^‐catalyzed labeling reaction. The biotinylation ratios were 93.7% on T cells and 89.2% on B cells respectively, demonstrating the generalizability of the method for studying microbe‐host cell interactions (Figure S12).

### 2.4 Combination of Bulk RNA‐Seq With the “Ru‐^1^O_2_‐Hydrazide” System to Analyze the Mechanism of the Interaction between *S. aureus* and HeLa Cells

**FIGURE 4:**
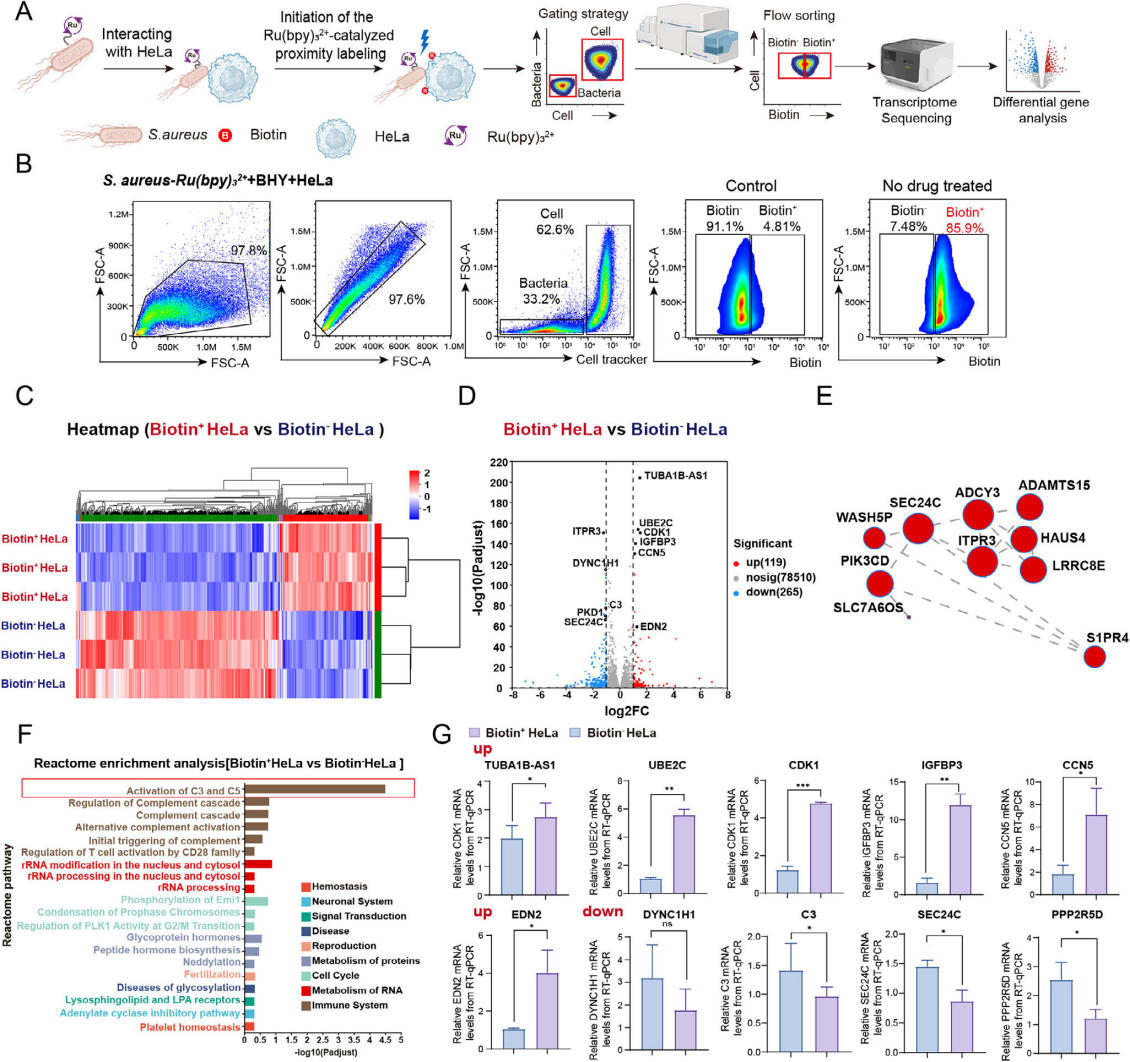
Use of the *S. aureus*‐*Ru(bpy)_3_^2+^* probe to identify *S. aureus*‐HeLa interactions and analyze the underlying mechanisms. (A) Schematic workflow for labeling and analyzing HeLa cells interacting with *S. aureus*. (B) Flow cytometry gating strategy for isolating Biotin+(interacting) and Biotin‐(bystander) HeLa cells via fluorescence‐activated cell sorting (FACS). The background is defined as the signal produced on HeLa after incubation with *S. aureus* without anchoring *Ru(bpy)_3_^2+^*. (C) Heatmap of differentially expressed genes between Biotin+ and Biotin‐ HeLa cells. n = 3. (D) Volcano plots of differentially expressed genes between Biotin+ and Biotin‐ HeLa cells. n = 3. (E) Correlation analysis of membrane proteins encoded differentially expressed genes between Biotin+ and Biotin‐ HeLa cells. n = 3. (F) Reactome enrichment analysis of the differentially expressed genes between Biotin+ and Biotin‐ HeLa cells. Bars represent the ‐log10(Padjust). n = 3. (G) RT‐qPCR analysis of *TUBA1B‐AS1*, *UBE2C*, *CDK1*, *IGFBP3*, *CCN5*, *EDN2*, *DYNC1H1*, *C3*, *SEC24C* and *PPP2R5D* expression levels highlighted in the volcano plot between Biotin+ and Biotin‐ HeLa. n = 3. (n: number of biological replicates. ns *p*>0.05; ^*^ *p*<0.05; ^**^ *p*<0.01; ^***^ *p*<0.001.).

When studying the interactions between *S. aureus* and HeLa cells using Ru(bpy)_3_^2+^‐catalyzed proximity labeling technology, the HeLa cells were divided into biotin‐positive and biotin‐negative groups. Our aim was to analyze the mechanisms of interaction between *S. aureus* and HeLa cells by investigating the differences between these two cell groups. We sorted the interacting (Biotin+) and bystander (Biotin‐) HeLa cells (Figure 4A,B). Bulk RNA‐seq analysis of these samples allowed us to compare their gene expression profiles (Figure 4C). Significantly, comparing to Biotin‐ HeLa cells, the Biotin+ HeLa cells subset was found to expressed high levels of *TUBA1B‐AS1* [45, 46]*, UBE2C* [47], *CDK1* [48], *IGFBP3* [49]*, CCN5, C1QL1* and so on, and low levels of ITPR3 [50], *GCN1* [51], *DYNC1H1* [52], *C3* [53], *PKD1* [54], *SEC24C* [55] and *PPP2R5D* (Figure 4D). The upregulated genes are primarily involved in regulating cancer cell proliferation and division (Table S1). Compared to their Biotin‐ counterparts, Biotin+ HeLa cells displayed transcriptional features associated with increased proliferation and malignancy, alongside reduced motility and impaired DNA damage recognition. These phenotypic traits were corroborated by pathway enrichment analyses. Reactome enrichment revealed that differentially expressed genes were primarily associated with complement activation (C3 and C5) (Figure 4F), with malignancy‐related genes showing elevated expression in the Biotin+ subset (Figure S13A). This transcriptional landscape suggests that HeLa cells interacting with *S. aureus* exhibit a more malignant phenotype and enhanced immune activation, consistent with host responses during pathogen infection [43, 44]. We also performed enrichment analysis on genes related to the immune system. Enrichment analyses using DO, KEGG, and GO terms further linked the differentially expressed genes to complement deficiency, *S. aureus* infection, and immune regulation, respectively (Figure S13B–D). Furthermore, RT‐qPCR validation confirmed that genes upregulated (*TUBA1B‐AS1*, *UBE2C*, *CDK1*, *IGFBP3*, *CCN5*, *EDN2*) or downregulated (*DYNC1H1*, *C3*, *SEC24C*, *PPP2R5D*) in Biotin+ HeLa cells were altered in agreement with the RNA‐seq analysis (Figure 4G). Additionally, we analyzed the differentially expressed genes encoding membrane proteins on the surfaces of Biotin+ and Biotin‐ HeLa cells. These membrane proteins are primarily involved in the cell cycle, proliferation, signal transduction, intercellular communication, substance transport, and metabolism (Table S2). Furthermore, our analysis revealed a correlation between these genes and their corresponding membrane proteins. This provides a basis for identifying the key protein molecules that mediate interactions between *S. aureus* and HeLa cells (Figure 4E). Collectively, these results indicate that the Biotin+ HeLa cell subset is characterized by transcriptional features associated with increased proliferative capacity and malignancy, as well as a phenotype that favors bacterial interaction.

### 2.5 Evaluation of Metal Ions in Modulating Bacteria‐Host Cell Interactions via the “Ru‐^1^O_2_‐hydrazide” System

**FIGURE 5:**
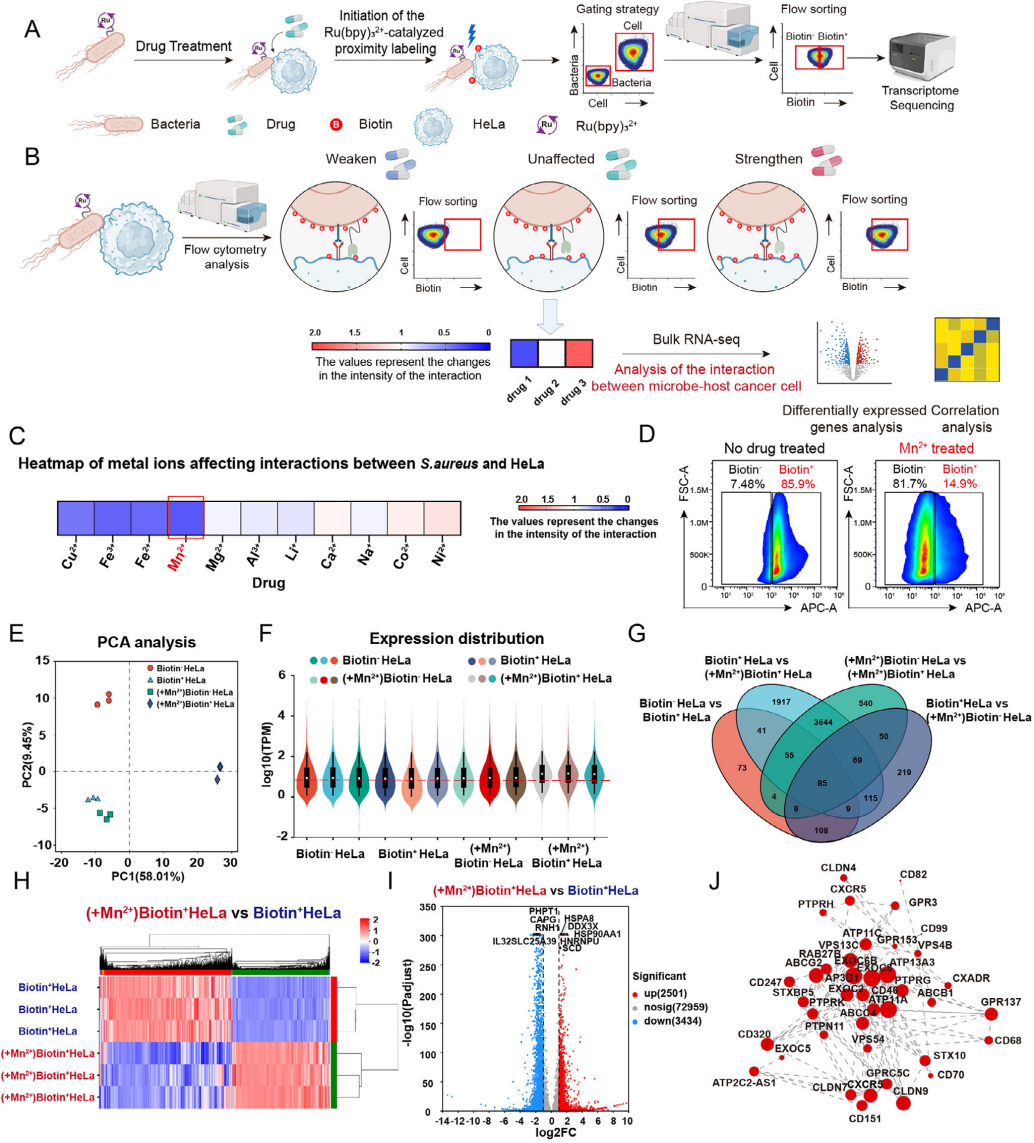
Evaluation of metal ions in modulating bacteria‐host cell interactions via the “Ru‐^1^O_2_‐hydrazide” system. (A, B) Schematic workflow for evaluating the effects mechanisms of different metal ions on the interaction between *S. aureus* and HeLa cells. (C) Flow cytometry gating strategy for isolating Biotin+ (no drug treatment group) and Biotin+(Mn^2+^ treatment group) HeLa cells via fluorescence‐activated cell sorting (FACS). (D) Heatmap representative of different metal ions affecting interaction between *S. aureus* and HeLa cells. The color blocks represent the intensity of the interaction between *S. aureus* and HeLa cells. n = 3. (E) PCA representative analysis of the group of Biotin+ HeLa, Biotin‐ HeLa, Biotin+ (Mn^2+^ treated group) HeLa, and Biotin‐ (Mn^2+^ treated group) HeLa. n = 3. (F) Violin diagram representative showing of the gene expression distribution in the group of Biotin+ HeLa, Biotin‐ HeLa, Biotin+ (Mn^2+^ treated group) HeLa, and Biotin‐ (Mn^2+^ treated group) HeLa. n = 3. (G) Venn diagram illustration of the number of differentially expressed genes found in Biotin‐ HeLa vs. Biotin+ HeLa, Biotin+ HeLa vs. Mn^2+^ treated Biotin+ HeLa, Mn^2+^ treated Biotin‐ HeLa vs. Mn^2+^ treated Biotin+ HeLa and Biotin+ HeLa vs. Mn^2+^ treated Biotin‐ HeLa. (H) Heatmap representative of differentially expressed genes between Biotin+ HeLa cells from the Mn^2+^ treated group and those from the untreated group. n = 3. (I) Volcano plots representative of differentially expressed genes between Biotin+ HeLa cells from the Mn^2+^ treated group and those from the untreated group. n = 3. (J) Correlation analysis of membrane proteins encoded by differentially expressed genes between Biotin+ HeLa cells from the Mn^2+^ treated group and those from the untreated group. n = 3.

Within the tumor microenvironment (TME), bacteria and host cancer cells engage in a complex and dynamic interplay that is not only fundamental to tumor biology but also central to emerging bacterium‐assisted anticancer strategies, such as bacterial‐targeted therapies [56, 57, 58, 59]. In this interaction, metal ions are far from passive bystanders; rather, they play critical roles as regulators and bridges. Investigating the influence and mechanisms of different metal ions (including Cu^2+^, Fe^3+^, Fe^2+^, Mn^2+^, Mg^2+^, Al^3+^, Li^+^, Ca^2+^, Na^+^, Co^2+^, and Ni^2+^) on this cross‐kingdom interaction is essential for its targeted modulation. First, after confirming via MTT assay and plate counting that both *S. aureus* and HeLa cells maintained >90% viability following treatment with 10 µm of each metal ion (Figure S14), we applied this concentration to the interacting co‐culture. Ru(bpy)_3_^2+^‐catalyzed proximity labeling was then employed to capture changes in their interaction and to evaluate the impact of the various metal ions (Figure 5A,B). We measured the biotinylation ratio of HeLa cells under different metal ion treatments and normalized these values relative to the metal‐free control. The resulting heatmap revealed that treatments with Cu^2+^, Fe^3+^, Fe^2+^, and Mn^2+^ all reduced the *S. aureus*–HeLa interaction (Figure 5C and Figures S15 and S16). The mechanisms by which Cu^2+^, Fe^3+^, and Fe^2+^ suppress *S. aureus*–HeLa interactions have been relatively well‐documented in previous research. This effect primarily stems from the dual modification of bacterial and cellular surfaces by metal ions, which alters the electrostatic attraction and specific molecular recognition processes between the two cell types. *S. aureus* initiates infection through initial electrostatic attraction between its negatively charged surface components (e.g., teichoic acids and carboxyl groups) and the negatively charged HeLa cell membrane (due to glycoproteins and phospholipids) [60]. Additionally, bacterial surface adhesins and fibronectin‐binding proteins (FnBPs) specifically recognize host cell receptors such as integrins and fibronectin, enabling robust adhesion [61, 62, 63, 64]. Metal ions interfere with this process through multiple mechanisms. First, cations (Cu^2+^, Fe^2+^ and Fe^3+^) can neutralize the negative charges on bacterial and cellular surfaces by charge shielding, thereby hindering the bacteria's ability to approach cells [65]. This reduces non‐specific adsorption and subsequent specific binding events. On the other hand, they can directly damage bacterial adhesins. Cu^2+^ oxidizes the sulphydryl groups of adhesins due to its strong oxidizing capacity, causing protein denaturation or inactivation [66]. Fe^2+^ generates reactive oxygen species (ROS) via the Fenton reaction, which oxidizes and damages adhesins and lipid structures indiscriminately. Although less oxidizing, Fe^3+^ can still cause conformational changes in binding proteins, indirectly impairing their function. Furthermore, these ions act directly on HeLa cells. ROS derived from Cu^2+^ and Fe^2+^ oxidatively damage cell surface receptors (e.g., integrins), thereby preventing bacterial recognition and binding [67]. Additionally, the metal ions physically shield receptor sites, akin to ‘armouring’ the cells, thereby blocking contact between bacterial ligands and receptors. In summary, Cu^2+^, Fe^2+^ and Fe^3+^ ions effectively inhibit the adhesion and invasion of *S. aureus* into HeLa cells through multiple physicochemical mechanisms (Table 1). However, few reports exist on how Mn^2+^ affects the interaction between *S. aureus* and HeLa cells and the underlying mechanisms. We combined the “Ru‐^1^O_2_‐hydrazide” system with transcriptome analysis to elucidate the mechanism by which Mn^2+^ influences this interaction. Metal immunology, an emerging field of interest, holds significant promise for elucidating the crucial regulatory functions of metal ions in immune responses [68, 69, 70]. Upon Mn^2+^ treatment, the biotinylation ratio of Biotin+ HeLa cells changed from 85.9% to 14.9%, indicating that the interaction between *S. aureus* and HeLa cells was decreased (Figure 5D). We sorted the interacting (Biotin+) and bystander (Biotin‐) HeLa cells and compared them with Biotin+ HeLa and Biotin‐ HeLa cells in the absence of Mn^2+^ treatment. Bulk RNA‐seq analysis of these samples allowed us to compare their gene expression profiles. First, we performed Principal Component Analysis (PCA), violin analysis, and heatmap on the sequencing results of Biotin+ and Biotin‐ HeLa cells from the Mn^2+^ treatment group and the untreated control group, revealing good parallelism between replicates (Figure 5E–H). Volcano plot analysis revealed that differentially expressed genes were associated with cancer cell proliferation and progression. Upregulated genes such as *HSPA8, DDX3X, HSP90AA1, HNRNPU*, and *SCD* and downregulated genes such as *PHPT1, GAPG, RNH1, IL32*, and *SLC25A39* have been previously implicated in these processes [71, 72, 73, 74, 75, 76, 77, 78] (Figure 5I). These findings suggest that Mn^2+^ treatment may suppress proliferative and migratory capacities in cancer cells.

**TABLE 1:** Summary of the mechanisms by which Cu^2+^, Fe^3+^, Fe^2+^ reduce the *S. aureus*–HeLa interactions.

Metal ion	Differences	Commonalities
Cu^2+^	1. Oxidizes sulphydryl groups of adhesins due to its strong oxidizing capacity, causing protein denaturation or inactivation [66]. 2. ROS derived from it oxidatively damages cell surface receptors (e.g., integrins) [67].	1. The dual modification of bacterial and cellular surfaces by metal ions which alters the electrostatic attraction and specific recognition processes between two types of cells. 2. Neutralize negative charges on bacterial and cellular surfaces by shielding [60], hindering bacterial approach to cells and reducing non‐specific adsorption and subsequent specific binding events [65]. 3. Interfere with bacterial adhesion tools or host cell receptors, preventing bacterial recognition and binding to host cells [61, 62, 63, 64].
Fe^2+^	1. Generates reactive oxygen species (ROS) via the Fenton reaction, oxidizing and damaging adhesins and lipid structures indiscriminately [67]. 2. ROS derived from it oxidatively damages cell surface receptors [67].	
Fe^3+^	1. Causes conformational changes in binding proteins (though less oxidizing), indirectly impairing their function [67].	

Further functional enrichment analyses supported this interpretation. Reactome enrichment analysis suggested that the differentially expressed genes were mainly enriched in cellular responses to stimuli and cellular responses to stress. (Figure S17A). GO enrichment analysis suggested that the differentially expressed genes were mainly enriched in membrane‐bounded organelle (Figure S17B); DO enrichment analysis suggested that the differentially expressed genes were mainly enriched in telangiectasis and velocardiofacial syndrome (Figure S17C) and KEGG enrichment analysis revealed that the differentially expressed genes were mainly enriched in ribosome, cell senescence, infection, and so on (Figure S17D). The presence of both upregulated and downregulated genes suggests that Biotin+ HeLa cells in the Mn^2+^‐treated group exhibited slower proliferation and malignancy progression compared to those in the untreated group. Additionally, we analyzed the expression of differentially expressed genes encoding membrane proteins on the surfaces of Biotin+ HeLa cells in the Mn^2+^ treatment group compared to those in the untreated group. These membrane proteins are primarily involved in signal transduction and cellular communication, cell adhesion and connections, and vesicular trafficking and membrane (Table S3). Furthermore, our analysis revealed a correlation between these genes and their corresponding membrane proteins, which play an important role in the interaction (Figure 5J).

**FIGURE 6:**
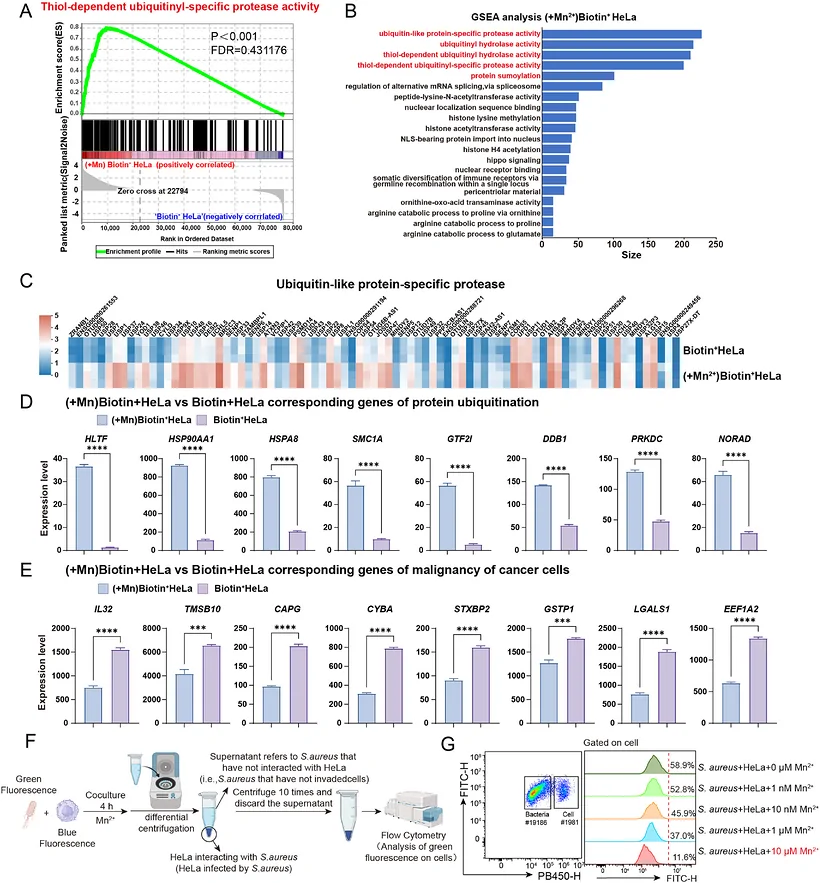
Analysis of the effects of Mn^2+^ on the interaction between *S. aureus* and HeLa cells. (A) GSEA showing enrichment of ubiquitin‐like protein‐specific protease activity in Biotin+ HeLa cells with Mn^2+^ treatment. All nominal *p* values < 0.001 and FDR < 0.5. Expanded GSEA results are presented in Figure S18A–D. (B) GSEA was performed based on the GO database. GO terms for the important functions are highlighted in red. (C) Heatmap generated from GSEA using the GO database. (D, E) Bar chat showing the expression levels of the genes associated with protein ubiquitination and cancer cell malignancy in Mn^2+^ treated vs. untreated Biotin+ HeLa cells. n = 3. (F, G) Schematic workflow and flow cytometry analysis of Mn^2+^ mediated inhibition of *S. aureus* invasion into HeLa cells. (n: number of biological replicates. ^***^ *p*<0.001; ^****^ *p*<0.0001.).

While this experiment was conducted using a cell line model, the impact of Mn^2+^ supplementation on the interactions between *S. aureus* and host cancer cells, and its effect on Biotin+ HeLa cell populations should not be underestimated. We also conducted a Gene Set Enrichment Analysis (GSEA) to identify pathways enriched in Biotin+ HeLa cells associated with thiol‐dependent ubiquitin hydrolase activity (Figure 6A–C and Figure S18A–H). Additionally, we analyzed Biotin+ HeLa cells that had been treated with Mn^2+^, as well as untreated Biotin+ HeLa cells. We examined cancer malignancy‐associated genes, including *IL32* [79]*, TMSB10* [80], *CAPG* [81], *CYBA* [82], *STXBP2* [83], *GSTP1* [84], *LGALS1* [85], *EEF1A2* [86], *MSLN* [87], *FKBP8* [88], *HSPB1* [89], *CA9* [90]*, UBE2S* [91], *FOLR1* [92], *PRDX5* [93], *BST2* [94]*, H19* [95], *SLC6A8* [96], *NIBAN2* [97], as well as ubiquitin‐related genes, inculding *HLTF* [98], *HSP90AA1* [99], *HSPA8* [100]*, SMC1A* [101]*, GTF2I* [102], *DDB1* [103], *PRKDC* [104], *NORAD* [105] (Tables S4 and S5). We found that in Mn^2+^ treated Biotin+ HeLa cells, the cancer malignancy‐associated genes were downregulated, whereas the ubiquitin‐related genes were upregulated. This suggests that Mn^2+^ reversed the malignant properties of HeLa cells (Figure 6D,E and Figure S19A). During tumor progression, the ubiquitination of numerous key proteins affects oncogenesis and tumor development [106, 107, 108, 109]. Here, we show that Mn^2^^+^ inhibits *S. aureus* invasion of HeLa cells in a dose‐dependent manner, as evidenced by reduced bacterial adhesion in flow cytometry assays (Figure 6F,G). This diminished interaction may contribute to suppressing cancer cell progression, which is consistent with previously reported findings [110]. These findings highlight the value of proximity labeling strategies for identifying critical molecular alterations in specific cancer cell subsets, offering a promising avenue for discovering novel therapeutic targets.

Studying the functions of metal ions in the interaction between bacteria and host cancer cells is key to deeply understanding the complexity of the tumor microenvironment from a novel “chemical biology” perspective [111, 112]. This approach not only answers fundamental scientific questions but also holds great potential for translational medicine. It promises to bring breakthrough strategies for cancer diagnosis, prevention, and treatment (especially bacterial‐based therapies), and ultimately promoting the development of precision medicine and new anti‐tumor drugs [113, 114, 115, 116].

## 3 Discussion

In summary, we employed a photocatalytic proximity labeling method‐the “Ru‐^1^O_2_‐hydrazide” system based on ruthenium complexes to capture the interactions between bacteria and host cancer cells. This system does not rely on genetic manipulations and is thus readily applicable to any type of bacteria, even primary pathogens. We used the [Ru(bpy)_3_^2+^]2PF_6_^−^ to construct different bacteria‐based probes, such as *S. aureus‐Ru(bpy)_3_^2+^* and *E. coli‐Ru(bpy)_3_^2+^*, demonstrating its general applicability. Featuring easy preparation and ready‐to‐adopt protocols, the “Ru‐^1^O_2_‐hydrazide” system serves as an attractive approach for the precise capture and quantification of bacteria‐host cancer cell interactions under simulated physiologically relevant conditions by providing resolved molecular information.

Utilizing the *S. aureus*‐*Ru(bpy)_3_^2+^* probe, we successfully captured diverse bacteria‐host cancer cell interactions without requiring prior knowledge of their molecular mechanisms. By integrating this proximity labeling approach with bulk RNA‐seq, we demonstrated that interaction with *S. aureus* enhances HeLa cell proliferation, malignancy, and surprisingly, immune‐related functions. Furthermore, leveraging the quantitative capability of the “Ru‐^1^O_2_‐hydrazide” system, we identified Mn^2+^ as a significant inhibitor of *S. aureus*‐HeLa interactions. Transcriptomic analysis revealed that Mn^2^^+^ attenuates the malignant phenotype of HeLa cells and impedes bacterial invasion, thereby weakening the interaction.

Our findings establish the “Ru‐^1^O_2_‐hydrazide” system as an effective strategy for capturing and quantifying microbe‐host cell interactions. This capability is particularly relevant given mounting evidence that such interactions promote tumorigenesis, progression, and metastasis [117, 118, 119, 120, 121, 122, 123]. This system offers a valuable paradigm for screening therapeutic agents that disrupt these pathogenic contacts. By enabling the quantitative assessment of interaction strength, it facilitates the discovery and mechanistic investigation of potential drugs.

Looking forward, combining the “Ru‐^1^O_2_‐hydrazide” system with advanced single‐bacterium omics technologies, such as cellular indexing of transcriptomes and epitopes (CITE‐seq), could further resolve the spatial heterogeneity of molecular determinants governing bacteria‐host interactions [21, 124]. Indeed, as several studies have highlighted drugs that modulate microbe‐host dynamics within the tumor immune microenvironment to suppress tumor progression, we anticipate that the “Ru‐^1^O_2_‐hydrazide” system will find broad applicability in evaluating the efficacy of such candidate therapeutics and accelerating anticancer drug discovery.

## Author Contributions

S. Q., X. H. Z, and X. L. designed the experimental strategies and wrote the manuscript. A. M. S., K. H. W., H. F. S., and K. M. T. performed the experiments. S. Q. and A. M. S. prepared the figures, analyzed the data, and edited the manuscript. All authors contributed to the preparation of the manuscript. S. Q. is the lead contact.

## Conflicts of Interest

The authors declare no conflicts of interest.

## Supporting information




**Supporting File 1**: advs76240‐sup‐0001‐SuppMat.docx.

**Supporting File 2**: advs76240‐sup‐0002‐SuppMat.docx.

## Data Availability

The data that support the findings of this study are available from the corresponding author upon reasonable request.
