# Supplementary material for: Use of the “Ru‐1O2‐Hydrazide” System Catalyzed by Metallic Ruthenium Complexes to Decipher the Interaction Between Microbes and Host Cancer Cells
Source: Adv Sci (Weinh). 2026 Jun 22;13(52):e76240. doi: 10.1002/advs.76240 (PMC13336812; doi:10.1002/advs.76240)
Supplement: Supplementary file 1 [file ADVS-13-e76240-s001.docx]

**Figure S1**

**Figure S2**

**Figure S3**

**Figure S4**

**Figure S5**

**Figure S6**

**Figure S7**

**Figure S8**

**Figure S9**

**Figure S10**

**Figure S11**

**Figure S12**

**Figure S13**

**Figure S14**

**Figure S15**

**Figure S16**

**Figure S17**

**Figure S18**

**Figure S19**

| **Gene ID** | **Gene Name** | **Gene Function** |
| --- | --- | --- |
| ENSG00000258017 | *TUBA1B-AS1* | As a prognostic and immunobiological marker of cancer in both pancreatic and breast cancers, and has been shown to mediate cancer cell growth^45,46^. |
| ENSG00000175063 | *UBE2C* | Encoding a key enzyme that initiates the labeling process which triggers cell division, thereby acting as a central regulator of mitosis^47^. |
| ENSG00000170312 | *CDK1* | Serves crucial regulator of cell mitosis; complexes with Cyclin B and acts as a downstream substrate for serine/threonine protein kinase phosphorylation^48^. |
| ENSG00000146674 | *IGFBP3* | Encoding a protein that acts as a molecular dispatcher that regulates the expression of the growth factor IGF, thereby controlling cell growth or death^49^. |
| ENSG00000096433 | *ITPR3* | A downregulated gene; mediate calcium ion release from the endoplasmic reticulum^50^. |
| ENSG00000089154 | *GCN1* | Regulation of the initiation of cellular stress responses^51^. |
| ENSG00000197102 | *DYNC1H1* | Controls the dynein motor complex, the complex is responsible for retrograde transport in the cell centre^52^. |
| ENSG00000125730 | *C3* | Regulation of tumor metastasis^53^. |
| ENSG00000008710 | *PKD1* | Regulation of key target proteins associated with cancer therapy^54^. |
| ENSG00000176986 | *SEC24C* | Downregulation of this gene enhances T-cell activation and anti-tumor immunity^55^. |

**Table S1**

**List of all differentially genes (DEGs) related to cell membrane proteins between Biotin+ HeLa and Biotin- HeLa cells**

| **I. Cell Cycle and Proliferation** | | | | | | |
| --- | --- | --- | --- | --- | --- | --- |
| Gene ID | Gene names | Gene description | FC(LC/control) | P value | Significant | Regulate |
| ENSG00000170312 | *CDK1* | Cell cycle control (G2/M transition, mitosis) | 2.903228169 | 1.08E-154 | Yes | up |
| ENSG00000092036 | *HAUS4* | Microtubule spindle assembly (HAUS complex) | 0.464637678 | 2.05E-37 | Yes | down |
| ENSG00000166106 | *ADAMTS15* | Extracellular matrix proteolysis, angiogenesis | 0.364931098 | 3.25E-09 | Yes | down |
| **II. Signal Transduction and Cellular Communication** | | | | | | |
| ENSG00000166428 | *PLD4* | Phospholipid metabolism, immune regulation | 4.767483188 | 7.21E-20 | Yes | up |
| ENSG00000125910 | *S1PR4* | Sphingosine-1-phosphate receptor, immune cell migration | 2.446134423 | 4.72E-08 | Yes | up |
| ENSG00000231738 | *TSPAN19* | Tetraspanin family, cell membrane organization | 2.234960545 | 0.000935472 | Yes | up |
| ENSG00000258644 | *SYNJ2BP-COX16* | RNA gene (function unclear), potentially mitochondrial | 9.544008687 | 0.002505527 | Yes | up |
| ENSG00000096433 | *ITPR3* | Calcium release from endoplasmic reticulum | 0.43630929 | 9.6E-155 | Yes | down |
| ENSG00000124181 | *PLCG1* | Phospholipase signaling, calcium pathway activation | 0.470781354 | 2.36E-41 | Yes | down |
| ENSG00000171608 | *PIK3CD* | Phosphoinositide 3-kinase, cell growth signaling | 0.46829067 | 1.94E-08 | Yes | down |
| ENSG00000248592 | *STIMATE-MUSTN1* | STIM modulator (calcium signaling), muscle development | 0.466316089 | 0.000763649 | Yes | down |
| **III. Substance Transport and Metabolism** | | | | | | |
| ENSG00000227039 | *ITGB2-AS1* | ITGB2-AS1 is a long non-coding RNA that primarily acts as an oncogene. | 2.846487936 | 0.0000455 | Yes | up |
| ENSG00000176986 | *SEC24C* | COPII vesicle cargo sorting (ER export) | 0.479165355 | 3.69E-69 | Yes | down |
| ENSG00000292982 | *WASH5P* | Non-coding RNA (function unclear, pseudogene) | 0.402225421 | 1.1E-37 | Yes | down |
| ENSG00000138031 | *ADCY3* | cAMP synthesis, G-protein signaling | 0.491043322 | 2.71E-37 | Yes | down |
| ENSG00000124574 | *ABCC10* | Multidrug resistance transporter | 0.467699364 | 2.78E-15 | Yes | down |
| ENSG00000103061 | *SLC7A6OS* | Amino acid transporter subunit (y+LAT complex) | 0.483237763 | 2.24E-13 | Yes | down |
| ENSG00000171017 | *LRRC8E* | Volume-regulated anion channel (VRAC) subunit | 0.484163859 | 6.23E-12 | Yes | down |

**Table S2**

**List of all differentially genes (DEGs) related to cell membrane proteins between Biotin+ HeLa with Mn^2+^ treatment and Biotin+ HeLa cells with no treatment**

| **I. Signal Transduction and Cellular Communication** | | | | | | |
| --- | --- | --- | --- | --- | --- | --- |
| Gene ID | Gene names | Gene description | FC(LC/control) | P value | Significant | Regulate |
| ENSG00000117335 | *CD46* | Complement regulator/pathogen receptor | 2.236080975 | 3.90E-283 | Yes | up |
| ENSG00000179295 | *PTPN11* | Encodes SHP-2 phosphatase, regulates cell signaling (e.g., RAS/MAPK). | 2.551451779 | 2.44E-103 | Yes | up |
| ENSG00000133657 | *ATP13A3* | Polyamine transporter, important for cardiovascular development. | 2.586932187 | 7.54E-80 | Yes | up |
| ENSG00000068650 | *ATP11A* | Lipid flippase; maintains plasma membrane asymmetry. | 2.543051402 | 5.68E-58 | Yes | up |
| ENSG00000152894 | *PTPRK* | Receptor phosphatase; involved in cell adhesion. | 2.556354522 | 2.75E-30 | Yes | up |
| ENSG00000154639 | *CXADR* | Primary receptor for coxsackievirus and adenovirus. | 2.009565824 | 3.29E-28 | Yes | up |
| ENSG00000125257 | *ABCC4* | Drug transporter (MRP4); effluxes nucleotides and drugs. | 2.441002488 | 1.88E-27 | Yes | up |
| ENSG00000085563 | *ABCB1* | Drug transporter (P-gp); confers multidrug resistance. | 3.555766571 | 1.25E-18 | Yes | up |
| ENSG00000101974 | *ATP11C* | Lipid flippase for phosphatidylserine; important for B-cells. | 2.008595473 | 4.63E-14 | Yes | up |
| ENSG00000144724 | *PTPRG* | Tumor suppressor phosphatase; regulates cell growth. | 3.601998401 | 0.000000315 | Yes | up |
| ENSG00000198821 | *CD247* | Signaling subunit of the T-cell receptor (TCR). | 5.3032464 | 0.00000271 | Yes | up |
| ENSG00000118777 | *ABCG2* | Drug transporter (BCRP); protects cells from toxins. | 2.398634691 | 0.00000768 | Yes | up |
| ENSG00000261286 | *ATP2C2-AS1* | Long non-coding RNA; function under investigation. | 2.497443566 | 0.020424473 | Yes | up |
| ENSG00000167775 | *CD320* | Receptor for vitamin B12 (cobalamin) uptake. | 0.284787754 | 6.47E-185 | Yes | down |
| ENSG00000129226 | *CD68* | Scavenger receptor; marker for macrophages and monocytes. | 0.135325865 | 7.55E-175 | Yes | down |
| ENSG00000173264 | *GPR137* | Orphan G-protein coupled receptor; function not fully characterized. | 0.360421814 | 5.11E-57 | Yes | down |
| ENSG00000170412 | *GPRC5C* | Retinoic acid-inducible GPCR; role in cell adhesion and differentiation. | 0.363916275 | 4.32E-43 | Yes | down |
| ENSG00000160683 | *CXCR5* | Chemokine receptor for CXCL13; guides B-cells to lymphoid follicles. | 0.000138663 | 2.05E-27 | Yes | down |
| ENSG00000178623 | *GPR35* | GPCR activated by kynurenic acid; involved in immune and metabolic processes. | 0.300784424 | 6.04E-27 | Yes | down |
| ENSG00000080031 | *PTPRH* | Receptor-type phosphatase; putative tumor suppressor. | 0.411320726 | 3.7E-15 | Yes | down |
| ENSG00000181773 | *GPR3* | GPCR that constitutively activates signaling; involved in maintaining meiotic arrest. | 0.368279054 | 6.3E-13 | Yes | down |
| ENSG00000158292 | *GPR153* | Orphan GPCR; predicted role in neurotransmission. | 0.438955768 | 8.56E-13 | Yes | down |
| ENSG00000105204 | *DYRK1B* | Serine/threonine kinase; regulates insulin signaling and cell proliferation. | 0.275807116 | 0.001232033 | Yes | down |
| ENSG00000125726 | *CD70* | Ligand for CD27; costimulatory signal for T-cell and B-cell activation. | 0.179703155 | 0.023102051 | Yes | down |
| **II: Cell Adhesion and Connections** | | | | | | |
| ENSG00000177697 | *CD151* | Tetraspanin protein; regulates cell adhesion, migration and signal transduction. | 0.352246977 | 1.31E-219 | Yes | down |
| ENSG00000189143 | *CLDN4* | Claudin 4; component of tight junctions, regulates paracellular permeability. | 0.321704925 | 1.68E-121 | Yes | down |
| ENSG00000181885 | *CLDN7* | Claudin 7; component of tight junctions, crucial for epithelial integrity. | 0.406114583 | 1.09E-115 | Yes | down |
| ENSG00000002586 | *CD99* | Involved in leukocyte migration, T-cell adhesion and differentiation. | 0.487584649 | 1.37E-30 | Yes | down |
| ENSG00000085117 | *CD82* | Tetraspanin protein; metastasis suppressor, regulates cell motility and signaling. | 0.487691873 | 2.37E-10 | Yes | down |
| ENSG00000213937 | *CLDN9* | Claudin 9; component of tight junctions, important in ion selectivity. | 0.290258272 | 0.0000594 | Yes | down |
| **III: Vesicular Trafficking and Membrane** | | | | | | |
| ENSG00000132842 | *AP3B1* | Adaptor protein complex subunit (vesicle formation) | 2.067333808 | 2.03E-55 | Yes | up |
| ENSG00000119541 | *VPS4B* | ESCRT complex ATPase (membrane scission) | 2.727388593 | 3.52E-38 | Yes | up |
| ENSG00000144036 | *EXOC6B* | Exocyst complex component (vesicle tethering) | 2.334363519 | 4.76E-36 | Yes | up |
| ENSG00000070367 | *EXOC5* | Exocyst complex component (vesicle docking) | 2.07274167 | 7.73E-30 | Yes | up |
| ENSG00000129003 | *VPS13C* | Lipid transfer protein (membrane contact sites) | 2.738593754 | 1.453435271 | Yes | up |
| ENSG00000116903 | *EXOC8* | Exocyst complex component (vesicle transport) | 2.168081763 | 5.92E-28 | Yes | up |
| ENSG00000112685 | *EXOC2* | Exocyst complex component (vesicle tethering) | 2.279722391 | 3.59E-24 | Yes | up |
| ENSG00000041353 | *RAB27B* | Small GTPase (secretory vesicle regulation) | 2.8064272 | 7.83E-23 | Yes | up |
| ENSG00000143952 | *VPS54* | GARP complex subunit (retrograde transport) | 2.120835157 | 2.33E-20 | Yes | up |
| ENSG00000138190 | *EXOC6* | Exocyst complex component | 2.144183727 | 1.07E-14 | Yes | up |
| ENSG00000104915 | *STX10* | Syntaxin, regulates the fusion of neurotransmitter vesicles with the presynaptic membrane. | 0.337098719 | 3.38E-116 | Yes | down |
| ENSG00000105649 | *RAB3A* | Rab GTPase, regulates exocytosis-endosome fusion | 0.321143598 | 2.37E-20 | Yes | down |
| ENSG00000106089 | *STX1A* | Rab GTPase, regulates the docking and fusion of neurotransmitter/hormone vesicles. | 0.349165683 | 1.28E-16 | Yes | down |
| ENSG00000179044 | *EXOC3L1* | Exocyst complex-associated protein, regulates epithelial secretion and tumor metastasis. | 0.140817362 | 0.00465496 | Yes | down |

**Table S3**

| **Gene ID** | **Gene Name** | **Gene Function** |
| --- | --- | --- |
| ENSG00000008517 | *IL32* | Proinflammation and procancer. Creating an inflammatory environment in the tumor microenvironment activates pathways such as NF-κB, which is associated with the progression of pancreatic cancer, lung cancer, and other cancers^79^. |
| ENSG00000034510 | *TMSB10* | They are highly expressed in liver cancer and colorectal cancer, and promote proliferation, migration and inhibit apoptosis by regulating the cytoskeleton^80^. |
| ENSG00000042493 | *CAPG* | It remodels the cytoskeleton and enhances the motility of cancer cells^81^. |
| ENSG00000051523 | *CYBA* | As a component of NADPH oxidase, it generates reactive oxygen species (ROS) that act as signaling molecules to promote tumor growth^82^. |
| ENSG00000076944 | *STXBP2* | It is involved in vesicular transport and drives tumor growth by influencing specific signaling pathways in certain cancers^83^. |
| ENSG00000084207 | *GSTP1* | It induces tumor drug resistance by metabolizing and eliminating chemotherapeutic drugs^84^. |
| ENSG00000100097 | *LGALS1* | It is highly expressed in a variety of cancers and drives malignant progression by promoting angiogenesis and inhibiting T-cell function^85^. |
| ENSG00000101210 | *EEF1A2* | It is highly expressed in ovarian cancer, breast cancer, and other cancers. It not only undertakes translational functions but also activates pro-survival pathways such as PI3K/Akt, thereby promoting cancer cell proliferation and metastasis^86^. |
| ENSG00000102854 | *MSLN* | It is highly expressed in mesothelioma, pancreatic cancer, and ovarian cancer, promoting proliferation, migration, and invasion while inhibiting apoptosis. It is a promising target for immunotherapy and targeted therapy^87^. |
| ENSG00000105701 | *FKBP8* | It protects cancer cells from apoptosis by inhibiting the Bcl-2/Bax pathway, and is associated with chemoresistance and poor prognosis in a variety of cancers^88^. |
| ENSG00000106211 | *HSPB1* | As a molecular chaperone, it protects cancer cells from stress-induced death and strongly promotes invasion, metastasis, and treatment resistance^89^. |
| ENSG00000107159 | *CA9* | It is highly expressed in various solid tumors such as renal cell carcinoma, induced by HIF-1α, and regulates pH balance to promote tumor growth, invasion, and metastasis^90^. |
| ENSG00000108106 | *UBE2S* | It drives cell cycle progression by mediating the degradation of target proteins, and its high expression is associated with genomic instability and poor prognosis^91^. |
| ENSG00000110195 | *FOLR1* | It is highly expressed in various epithelial cancers such as ovarian cancer and lung cancer, serves as key target for tumor-targeted therapy, and promotes folate uptake and proliferation of cancer cells^92^. |
| ENSG00000126432 | *PRDX5* | As a peroxidase, it scavenges reactive oxygen species (ROS), protects cancer cells from oxidative stress-induced damage, and promotes their survival, chemoresistance, and metastasis. Its role varies across different types of cancer, but most studies have demonstrated its pro-cancer properties^93^. |
| ENSG00000130303 | *BST2* | It is highly expressed in a variety of cancers and promotes proliferation, invasion, and metastasis by activating signaling pathways such as NF-κB. It is also used as biomarker for certain cancers^94^. |
| ENSG00000130600 | *H19* | It strongly promotes cancer cell proliferation, metastasis, and treatment resistance, and is associated with the properties of cancer stem cells^95^. |
| ENSG00000130821 | *SLC6A8* | It is responsible for transporting creatine into cells, providing rapidly regenerated ATP for cancer cells with high energy demands, and directly supporting their proliferation, invasion and survival^96^. |
| ENSG00000136830 | *NIBAN2* | It significantly enhances the viability, treatment resistance, and metastatic potential of cancer cells  by inhibiting apoptosis, promoting autophagy, and adapting to stressful environments, and is associated with poor prognosis in a variety of cancers^97^. |

**Table S4**

| **Gene ID** | **Gene Name** | **Gene Function** |
| --- | --- | --- |
| ENSG00000071794 | *HLTF* | It possesses SWI/SNF chromatin remodeling activity and E3 ubiquitin ligase activity, and is involved in DNA damage repair. It is often silenced due to promoter methylation, with a function similar to that of a tumor suppressor gene^98^. |
| ENSG00000080824 | *HSP90AA1* | It is the major isoform of HSP90, responsible for stabilizing hundreds of "client proteins", many of which are key oncoproteins and kinases. Cancer cells are highly dependent on it to maintain protein stability and rapid proliferation, making it a crucial anti-cancer target^99^. |
| ENSG00000109971 | *HSPA8* | It is involved in protein folding, trafficking and autophagy. Highly expressed in cancer cells, it acts by inhibiting apoptosis, promoting autophagy and stabilizing oncoproteins^100^. |
| ENSG00000072501 | *SMC1A* | Core subunit of the cohesin complex. Similar to RAD21, it is a key protein for chromosome segregate ion and DNA repair. Its dysfunction leads to genomic instability, a hallmark of cancer. It is highly expressed in various tumors and associated with proliferation and poor prognosis^101^. |
| ENSG00000263001 | *GTF2I* | Transcription factors and oncogenes. This gene serves as a crucial oncogenic driver, and somatic mutations in it are frequently detected, especially in thymomas^102^. |
| ENSG00000167986 | *DDB1* | DNA Damage Repair and Ubiquitin Ligase Adaptor. It is a core component of the CRL4 ubiquitin ligase complex, involved in nucleotide excision repair to respond to UV damage, and regulates the cell cycle, apoptosis, and metabolism^103^. |
| ENSG00000253729 | *PRKDC* | Catalytic subunit of DNA-PK. It is the core kinase in the non-homologous end joining (NHEJ) path way for DNA double-strand break repair^104^. |
| ENSG00000260032 | *NORAD* | Long non-coding RNA (lncRNA). It is a potent negative regulator of the DNA damage response. Acting as a molecular sponge, it binds to and sequesters PUMILIO proteins, preventing them from degrading pro-survival mRNAsepair^105^. |

**Table S5**

| **Gene name** | **Primer name** | **sequences (5' to 3')** |
| --- | --- | --- |
| **β-actin** | β-actin-F | TCCCTGGAGAAGAGCTACGA |
|  | β-actin-R | AGCACTGTGTTGGCGTACAG |
| **UBE2C** | UBE2C-F | CGAGTTCCTGTCTCTCTGCC |
|  | UBE2C-R | CAGCTCCTGCTGTAGCCTTT |
| **CDK1** | CDK1-F | AAACTACAGGTCAAGTGGTAGCC |
|  | CDK1-R | TCCTGCATAAGCACATCCTGA |
| **IGFBP3** | IGFBP3-F | GCGCCAGGAAATGCTAGTG |
|  | IGFBP3-R | GGAACTTGGGATCAGACACCC |
| **CCN5** | CCN5-F | TGTGCCCGACACCATGTACC |
|  | CCN5-R | CCACAGCCATCCAGCACCAG |
| **EDN2** | EDN2-F | TTGGACATCATCTGGGTGAA |
|  | EDN2-R | CTGTAGTGGCCCCTGTCTTG |
| **DYNC1H1** | DYNC1H1-F | TTGCGGCCCTATGGTGAAAT |
|  | DYNC1H1-R | GGATCATCTGCTCCACCTCG |
| **C3** | C3-F | ATACCAAAAGGACGCCCCTG |
|  | C3-R | CAAGGTGCCTTGGCCTTTTC |
| **SEC24C** | SEC24C-F | GTGTTGAAGAGTGATGTCCTG |
|  | SEC24C-R | GCTGGTGGTTCGGTAGTA |
| **PPP2R5D** | PPP2R5D-F | ATCTACGAGACGGAGCAT |
|  | PPP2R5D-R | TAGGACACGGATGAGGAA |
| **TUBA1B-AS1** | TUBA1B-AS1-F | AGTTGTGGTTCGGATAGC |
|  | TUBA1B-AS1-R | GTTGTCATCAGGCACCTA |

**Table S6**
