# Supplementary material for: Use of the “Ru‐1O2‐Hydrazide” System Catalyzed by Metallic Ruthenium Complexes to Decipher the Interaction Between Microbes and Host Cancer Cells
Source: Adv Sci (Weinh). 2026 Jun 22;13(52):e76240. doi: 10.1002/advs.76240 (PMC13336812; doi:10.1002/advs.76240)
Supplement: Supplementary file 2 [file ADVS-13-e76240-s002.docx]

**Use of the “Ru-^1^O_2_-hydrazide” system catalyzed by metallic ruthenium complexes to decipher the interaction between microbes and host cancer cells**

Amin Sun^1#^; Kaihong Wang^1^^,2,3#^; Haifu Sun^1^; Kemei Tao^4^; Xiang Li^3^*; Xiuhua Zhao^1,2^*; Shuang Qiu^1,2^*

1 Key Laboratory of Forest Plant Ecology, Northeast Forestry University, Ministry of Education, Harbin 150040, Heilongjiang, China

2 College of Food and Health, Northeast Forestry University, Harbin 150040, Heilongjiang, China

3. Hubei Province Key Laboratory of Biotechnology of Chinese Traditional Medicine, College of Health Science and Engineering, Hubei University, Wuhan, 430062, China.

4. Shenzhen GeneSeqTools Bio-Science & Technology Co., Ltd, Floor 5, Building B4, Shenchengtou & Zhongcheng Life science park Julong Mountain Street A, Pinshan District, Shenzhen, 518000, China

\# These authors contributed equally

*Correspondence e-mail: qs@nefu.edu.cn; xiuhuazhao@nefu.edu.cn; [lixiang199284@hubu.edu.cn](mailto:lixiang199284@hubu.edu.cn)

**Abstract:**

The dynamic interplay between bacteria and host cancer cells plays a critical role in tumor microenvironment modulation, bacterial pathogenesis, and potential oncotherapy applications. However, traditional methods often fail to capture transient or spatially restricted molecular interactions at the bacteria-cancer cell interface. Proximity labeling has emerged as a promising technology for capturing the interaction between bacteria and host-cancer cell. Photocatalytic proximity labeling is more efficient, faster and higher resolution in enzyme-catalyzed proximity labeling. Here, we employ photoactivatable proximity labeling technology—the “Ru-^1^O_2_-hydrazide” system to rapidly capture the interaction between bacteria and host cancer . This system is using the singlet-oxygen(^1^O_2_) mechanism with hydrazide biotin by anchoring the photosensitizer Ru(bpy)_3_^2+^ on the bacteria surface to efficiently and discriminatively capture the bacteria-host cancer cell interactions (BHIs). Furthermore, we established a quantitative strategy based on the “Ru-^1^O_2_-hydrazide” system to characterize bacteria-host cell interaction strength. This strategy allows for systematic evaluation of drug effects on BHIs, offering novel insight into pharmaceutical modulation of bacteria-host cancer cell crosstalk.

Content

[Experimental Procedures	3](#_Toc11106)

[General Information	3](#_Toc20501)

[Syntheic methods.	3](#_Toc10807)

[Supplementary Methods	4](#_Toc25804)

[1. Chemical synthesis of [Ru(bpy)₃²⁺]2PF_6_^-^	4](#_Toc13583)

[2. Ru(bpy)₃²⁺ loaded on bacteria surface assay	4](#_Toc29553)

[3. Ru(bpy)_3_^2+^-mediated cell surface biotinylation assay	5](#_Toc23869)

[4. Ru(bpy)_3_^2+^ covalent loading rather than adhesion to bacteria surfaces validation assay	5](#_Toc22960)

[5. Ru(bpy)_3_^2+^ affected bacteria growth assay	5](#_Toc31934)

[6. Detection of bacteria-host cancer cell interactions	5](#_Toc14481)

[7. Quantitative labeling	5](#_Toc2957)

[8. Selectivity labeling	5](#_Toc24302)

[9. Detection of *S. aureus*-splenocytes interactions	5](#_Toc8385)

[10. Evaluating the effect of different metal ions on *S. aureus*-HeLa interactions using the “Ru-^1^O_2_-hydrazide” system	5](#_Toc1173)

[11. The toxicity effect of different metal ions on the growth of *S. aureus*	6](#_Toc1436)

[12. The cytoxicity effect of different metal ions on the growth of HeLa cells	6](#_Toc17328)

[13. High-content imaging	6](#_Toc12774)

[14. Flow cytometry and cell sorting	6](#_Toc27170)

[15. RNA-seq analysis	6](#_Toc1098)

[16. Data evaluation	6](#_Toc19182)

[17. RNA extraction and quantitative RT-qPCR	6](#_Toc24219)

[18. Bacterial invasion into cells assay	7](#_Toc14329)

[19. Statistical analysis	7](#_Toc19743)

[20. ^1^H NMR, ^13^C NMR, ^31^P NMR and HRMS spectra of compounds	7](#_Toc20122)

[Supplementary Figures	9](#_Toc22869)

[Figure S1. Characterization of bacteria tethered with Ru(bpy)_3_^2+^ and the activity of Ru(bpy)_3_^2+^ loaded onto bacteria.	9](#_Toc21967)

[Figure S2. Verfication of the Ru(bpy)_3_^2+^ loading via covalent bonding rather than adhering to the bacteria surface.	10](#_Toc27604)

[Figure S3. Exploration of the effect of loading Ru(bpy)_3_^2+^ onto bacteria and its impact on their viability.	10](#_Toc8987)

[Figure S4. Verification of singlet oxygen mediated labeling reactions.	11](#_Toc32306)

[Figure S5. Verification of labeling induced by proximity effects between bacteria and host cancer cells.	12](#_Toc25433)

[Figure S6. Quantitative exploration of the “Ru-^1^O_2_-hydrazide” system for studying the interaction between *S. aureus* and HeLa cells.	13](#_Toc9493)

[Figure S7. Quantitative exploration of the “Ru-^1^O_2_-hydrazide” system for studying the interaction between *S. aureus* and HeLa cells.	14](#_Toc20221)

[Figure S10. Exploration of selectivity of the “Ru-^1^O_2_-hydrazide” system for studying the interaction between *S. aureus* and cell mixtures.	16](#_Toc31709)

[Figure S11. Exploration of selectivity of the “Ru-^1^O_2_-hydrazide” system for studying the interaction between *S. aureus* and cell mixtures.	17](#_Toc19878)

[Figure S12. Use of the “Ru-^1^O_2_-hydrazide” system to study *S. aureus* and primary cells from murine spleen.	17](#_Toc1669)

[Figure S13. Use of the *S. aureus-Ru(bpy)_3_^2+^* probe to identify *S. aureus*-HeLa interaction and analyze the underlying interaction mechanisms.	18](#_Toc27367)

[Figure S14. Effects of different metal ions on bacterial and cell viability.	19](#_Toc946)

[Figure S15. Use of *S. aureus-Ru(bpy)_3_^2+^* probe to identify the effects of different metal ions on *S. aureus*-HeLa interaction.	20](#_Toc20145)

[Figure S16. Use of *S. aureus-Ru(bpy)_3_^2+^* probe to identify the effects of different metal ions on *S. aureus*-HeLa interaction.	21](#_Toc14339)

[Figure S17. Evaluation of metal ions in modulating bacteria-host cell interactions via the “Ru-^1^O_2_-hydrazide” system.	21](#_Toc15821)

[Figure S18. Analysis of the effects of Mn^2+^ on *S. aureus*-HeLa interactions.	22](#_Toc17698)

[Figure S19. Analysis of the effects of Mn^2+^ on *S. aureus*-HeLa interactions. (A)	23](#_Toc2871)

[Supplementary Tables	23](#_Toc29228)

[Table S1. Functional list of genes highlighted in the volcano plot comparing Biotin+ and Biotin- HeLa cells.	23](#_Toc26982)

[Table S2. List of all differentially expressed genes (DEGs) encoding cell membrane proteins in Biotin+ versus Biotin- HeLa cells.	24](#_Toc14425)

[Table S3. List of differentially expressed genes (DEGs) encoding cell membrane proteins in Mn^2+^ treated and untreated Biotin+ HeLa cells.	24](#_Toc17094)

[Table S4. Functional list of downregulated genes associated with cancer malignancy by GSEA in Mn^2+^ treated versus untreated Biotin+ HeLa cells.	25](#_Toc15904)

[Table S5. Functional list of upregulated genes associated with ubiquitination by GSEA in Mn^2+^ treated versus untreated Biotin+ HeLa cells.	26](#_Toc15073)

[Table S6. Primer sequences used for q-PCR.	26](#_Toc31775)

[Author Contributions	26](#_Toc17943)

[Reference	26](#_Toc25225)

# Experimental Procedures

# General Information

## Syntheic methods.

**Materials and reagents.** All chemical reagents and solvents were obtained from Sigma-Aldrich and used without further purification. Tris(2,2’-bipyridyl)ruthenium NHS-ester (#161698-59-5) was purchased from GeneseqTools. APC Streptavidin (#B388666), PE anti-human CD29 (#303003), PB anti-mouse CD3 (#100213), FITC anti-mouse CD19 (#152403) were purchased from Biolegend. eBioscience Cell Proliferation Dye Fluor 450 (#65-0842-85), carboxyfluorescein succinimidyl ester (CFSE) (#C34570) and CellTracker CM-DiI Dye (#C7000) were purchased from Thermo Fisher.

**Cell lines.** Cell lines were all purchased from American Type Culture Collection or National Infrastructure of Cell line Resource, China. SKOV3 and HeLa cell lines were grown in DMEM (Dulbecco’s modified Eagle’s medium; GlutaMAX, Gibco) supplemented with 10% fetal bovine serum (FBS; Omega Scientific Inc.) and 1× penicillin-streptomycin (Gibco). Jurkat were grown in RPMI 1640 (DMEM, GlutaMAX, Gibco) supplemented with 10% FBS (Gibco) and 1× penicillin-streptomycin (Gibco). All cell cultures were incubated at 37°C under 5% CO_2_/95% air.

**Primary cells.** C57BL/6J mice were purchased from Changsheng Bio-technology Co., Ltd. Murine splenocytes were isolated from C57BL/6J mice. Spleen was aseptically removed, placed in a 6 cm dish containing 10 mL of RPMI 1640 medium, and gently ground using sterile syringe plungers or frosted glass slides. The cell suspension was transferred to a 15 mL conical tube and centrifuged at 450 ×g for 5 min at 4°C. After discarding the supernatant, the pellet was treated with 1 mL of red blood cell lysis buffer for 3 min at room temperature. Lysis was stopped by adding 9 mL of Dulbecco’s phosphate‑buffered saline (DPBS). The sample was centrifuged again at 450 ×g for 5 min at 4°C, and the splenocytes was resuspended in an appropriate volume of RPMI 1640 medium for proximity‑labeling experiments. All animal experiments were approved by the Animal Ethical and Welfare Committee of Northeast Forestry University. The present study was approved by the Ethics Committee of Northeast Forestry University in accordance with the Declaration of Helsinki (protocol 2025012).

# Supplementary Methods

## 1. **Chemical synthesis of [Ru(bpy)₃²⁺]2PF_6_**^-^

Compound **1** was synthesized according to reference **1**.

Synthesis of Compound **2**

RuCl_3_ ∙ 3H_2_O (0.65 g), anhydrous lithium chloride (0.7 g), and 2,2ʹ-bipyridine (0.8 g) were dissolved in DMF (12.5 mL), being allowed to react at 150 °C for 8 h under magnetic stirring. The solution was cooled down to room temperature and then, 50.0 mL of acetone was added. The mixture was placed at 4 °C for 12 h and the purple black precipitate was collected by filtering. The product was rinsed with ethyl ether for three times, dried at 50 °C for 12 h, to obtain compound 2. 1H NMR (600 MHz, DMSO-d6) δ 9.95 (d, J = 5.6 Hz, 2H), 8.64 (dd, J = 8.2, 1.3 Hz, 2H), 8.49 (dd, J = 8.2, 1.3 Hz, 2H), 8.07 (t, J = 7.8 Hz, 2H), 7.76 (td, J = 5.9, 2.9 Hz, 2H), 7.66 (t, J = 7.7 Hz, 2H), 7.48 (d, J = 5.7 Hz, 2H), 7.12 (t, J = 6.5 Hz, 2H).

Synthesis of Compound **3**

Compound 2 (520 mg, 1.00 mmol) in hot EtOH/H_2_O (30 mL/50 mL) was combined with compound 1 (385 mg, 1.50 mmol) in hot EtOH (100 mL), and flushed with nitrogen gas for 15 min. The mixture was refluxed for three days and then cooled to room temperature. The volume was reduced to about 60 mL and a solution of NH_4_PF_6_ (815 mg 5.00 mmol) in 10 mL water was added. The precipitate was filtered, washed with water (2 × 50 mL), diethyl ether (2 × 50 mL) and dried in vacuo to yield compound 3 (brown solid). 1H NMR (600 MHz, Methanol-d4) δ 8.67 (d, J = 8.0 Hz, 4H), 8.57 (d, J = 5.4 Hz, 2H), 8.09 (t, J = 7.7 Hz, 4H), 7.82 (dt, J = 12.2, 5.6 Hz, 4H), 7.61 (dd, J = 23.6, 5.6 Hz, 2H), 7.48 (p, J = 6.8, 6.3 Hz, 4H), 7.33 (dd, J = 20.3, 5.1 Hz, 2H), 2.88 (t, J = 7.6 Hz, 2H), 2.57 (s, 3H), 2.35 (t, J = 7.1 Hz, 2H), 2.01 (p, J = 7.0 Hz, 2H).

Synthesis of Compound **4**

Compound 3 (960 mg, 1.00 mmol) and N-hydroxysuccinimide (138 mg, 1.20 mmol) were dissolved in 60 mL dichloromethane (DCM) at 0 °C Then, dicyclohexylcarbodiimide (248 mg, 1.20 mmol) dissolved in DCM was added dropwise. After stirring at ambient temperature overnight, the precipitate was filtered and the filtrate was evaporated under reduced pressure to yield a dark orange solid. The crude product was purified by silica gel column chromatography (DCM : MeOH = 30 : 1) to give an orange solid 876 mg, yield 82.9 %.1H NMR (600 MHz, Acetone-d6) δ 8.83 (d, J = 8.1 Hz, 4H), 8.78 (d, J = 19.7 Hz, 2H), 8.23 – 8.20 (m, 4H), 8.06 (dt, J = 11.1, 6.1 Hz, 4H), 7.93 (d, J = 5.8 Hz, 1H), 7.87 (d, J = 5.8 Hz, 1H), 7.60 – 7.56 (m, 4H), 7.50 (dd, J = 5.8, 1.7 Hz, 1H), 7.43 (ddd, J = 5.8, 1.9, 0.8 Hz, 1H), 3.02 (dd, J = 8.8, 6.8 Hz, 2H), 2.91 (s, 4H), 2.74 (t, J = 7.2 Hz, 2H), 2.58 (s, 3H), 2.16 (p, J = 7.3 Hz, 2H).

## 2. Ru(bpy)₃²⁺ loaded on bacteria surface assay

A total of 1 million Escherichia coli (*E. coli*), Staphylococcus aureus (*S. aureus*) or Bacillus subtilis (*B. subtilis*) cells were resuspended in 100 μL DPBS buffer. Cells were treated with 0.001 μM, 0.01 μM, 0.1 μM, 1 μM, 10 μM, 100 μM, 200 μM [Ru(bpy)₃²⁺]2PF_6_^-^, followed by incubation on ice for 20 min, Ru(bpy)_3_^2+^-anchored *E. coli*, *S. aureus* or *B. subtilis* cells were washed twice with PBS and resuspended in FACS buffer (2% FBS in DPBS), cells were detected by flow cytometric analysis.

## 3. Ru(bpy)_3_^2+^-mediated cell surface biotinylation assay

100 μM biotin-hydrazide were added separately to Ru(bpy)_3_^2+^-bound *E. coli* cells，Ru(bpy)_3_^2+^-bound *S. aureus* cells, Ru(bpy)_3_^2+^-bound *B. subtilis* cells, cells were irradiated with 450 nm light for 5 min and washed three times with FACS buffer and then stained with SA-APC for 30 min on ice for flow cytometric analysis.

## 4. Ru(bpy)_3_^2+^ covalent loading rather than adhesion to bacteria surfaces validation assay

100 μM [Ru(bpy)₃²⁺]2PF_6_^-^ were treated in advance with 50 μL DPBS buffer for 20 minutes at room temperature and then added to 1 million *E. coli*, *S. aureus* or *B. subtilis* cells separately in DPBS buffer, Cells were treated with 100 μM [Ru(bpy)₃²⁺]2PF_6_^-^, followed by incubation on ice for 20 min, *E. coli*, *S. aureus* or *B. subtilis* cells were washed twice with PBS and resuspended in FACS buffer (2% FBS in DPBS), cells were detected by flow cytometric analysis.

## 5. Ru(bpy)_3_^2+^ affected bacteria growth assay

The same amount of Ru(bpy)_3_^2+^-bound *E. coli* cells and *E. coli* cells were applied to the agar plate that was suitable for their growth at 37℃ for 16 h. Then the number of bacteria on each of the two plates were checked.

## 6. Detection of bacteria-host cancer cell interactions

E. *coli* or *S. aureus* tethered with Ru(bpy)₃²⁺ were incubated with SKOV3 (HeLa, or Jurkat) cells at 37°C for 2 h at a 10:1 ratio. 100 μM biotin-hydrazide was added to the mixtures of bacteria and cells. The mixtures were irradiated with a 450 nm LED (30 mW, 3.31 mW/cm²) for 5 min at a distance of 12 cm. Mixtures were then centrifuged (450g, 5 min), washed three times with FACS buffer, and analyzed by flow cytometry.

## 7. Quantitative labeling

**Proximity labeling assay in an antibody blocking model**

HeLa cells were pretreated with CD29 antibody at concentrations of 1 μg/mL, 0.75 μg/mL, 0.5 μg/mL, 0.07 μg/mL, 0.05 μg/mL, and 0.01 μg/mL to block integrin α5β1 on the cell surface, which is involved in *S. aureus* adhesion. The *S. aureus-Ru(bpy)_3_^2+^* probe was then co-incubated with the antibody-pretreated HeLa cells at the same cell number and ratio. Subsequently, 100 μM biotin-hydrazide substrate was added, the illumination conditions, staining, washing, and flow cytometry analysis procedures for the bacteria-cell mixtures were the same as described above.

**Proximity labeling assay of bacterial adhesion to host cells**

At a co-incubation ratio of *S. aureus* to HeLa cells of 10:1, the degree of adhesion of *S. aureus* to HeLa cells was modulated by setting different co-incubation times (1, 2, 4, and 6 hours), thereby establishing a gradient model of *S. aureus*-HeLa interaction intensity. 100 μM biotin-hydrazide was added separately to the mixed systems of *S. aureus* or *S. aureus-Ru(bpy)_3_^2+^* with HeLa cells. The illumination conditions, staining, washing, and flow cytometry analysis procedures for the bacteria-cell mixtures were the same as described above.

By controlling the ratios of S. aureus to HeLa cells at 10:1, 5:1, 1:1, and 0.5:1, the degree of adhesion of *S. aureus* to HeLa cells was modulated, thereby establishing a gradient model of interaction intensity between *S. aureus* and HeLa cells. 100 μM biotin-hydrazide was added separately to the mixed systems of S. aureus or *S. aureus-Ru(bpy)_3_^2+^* with HeLa cells. The illumination conditions, staining, washing, and flow cytometry analysis procedures for the bacteria-cell mixtures were the same as described above.

## 8. Selectivity labeling

The prepared *E. coli-Ru(bpy)_3_^2+^* and *S. aureus-Ru(bpy)_3_^2+^* were added to cell mixtures of SKOV3&HeLa, SKOV3&Jurkat or HeLa&Jurkat (at the ratio of 1:1) and then incubated at 37° C for 2 h. 100 μM biotin-hydrazide were added separately to cell mixtures of *E. coli* or *S. aureus* tethered to Ru(bpy)_3_^2+^ and SKOV3 cells. Bacterial-cell mixture illumination conditions, staining, washing, and flow cytometric analysis procedures are the same as above.

## 9. Detection of *S. aureus*-splenocytes interactions

*S. aureus* tethered with Ru(bpy)_3_^2+^ were incubated with splenocytes at 37° C for 2 h. After washing three times with PBS, 100 μM biotin-hydrazide were added to the mixtures of *S. aureus* tethered to Ru(bpy)_3_^2+^ and splenocytes. *S. aureus* and splenocytes were co-incubated in a 96-well plate at a ratio of approximately 1:1. *S. aureus* and splenocytes mixtures were irradiated with a 450 nm wavelength light source at 30 mW power for five minutes. The light intensity is 3.31 mW/cm². The distance between the flat panel and the LED light is 12 cm. Washing the mixtures of bacteria and cells: centrifuge at 450g for five minutes using a plate centrifuge. Aspirate the supernatant, resuspend in 100 μL FACS, washed three times with FACS buffer and then stained for flow cytometric analysis.

## 10.  Evaluating the effect of different metal ions on *S. aureus*-HeLa interactions using the “Ru-^1^O_2_-hydrazide” system

The prepared *S. aureus-Ru(bpy)_3_^2+^* were incubated with HeLa cells at the ratio of 10:1, adding 10 μM CuCl_2_, FeCl_3_, FeCl_2_, MnCl_2_, AlCl_3_, KCl, LiCl, CaCl_2_, NaCl, CoCl_2_ and NiCl_2_ to bacteria-cancer cell mixtures and incubating at 37℃ for 4 h. 100 μM biotin-hydrazide were added separately to cell mixtures of *E. coli* or *S. aureus* tethered to Ru(bpy)_3_^2+^ and SKOV3 cells. Bacterial-cell mixture illumination conditions, staining, washing, and flow cytometric analysis procedures are the same as above.

## 11.  The toxicity effect of different metal ions on the growth of *S. aureus*

Add 10 μM of each of the following to the prepared *S. aureus*: CuCl₂, FeCl₃, FeCl₂, MnCl₂, AlCl₃, KCl, LiCl, CaCl₂, NaCl, CoCl₂ and NiCl₂. Incubate at 37 °C for 4 hours. The number of bacteria was then counted by flow cytometry. The bacteria were serially diluted with PBS buffer to a count of 2 and evenly spread on prepared solid agar plates. The plates were cultured at 37 °C for 16 hours and the number of colonies was subsequently counted.

## 12. The cytoxicity effect of different metal ions on the growth of HeLa cells

Transfer the digested HeLa cells from the culture flask to a 96-well plate at a density of approximately 2,000 cells per well. After the cells have attached, add 10 µM of each of the following: CuCl₂, FeCl₃, FeCl₂, MnCl₂, AlCl₃, KCl, LiCl, CaCl₂, NaCl, CoCl₂ and NiCl₂. Then incubate at 37 °C under 5% CO₂ for 4 hours. Add 10 μL of the prepared MTT solution to each well, then continue to incubate for a further four hours. Then, add 100 μL of methylated spirit dissolution solution to each well. Mix thoroughly and continue to incubate in the cell culture incubator until complete dissolution of the methylated spirit is observed under a standard optical microscope. The purple crystals typically dissolve completely after 3–4 hours of incubation at 37 °C. Measure the absorbance at 570 nm using a microplate reader.

## 13. High-content imaging

Harvest the SKOV3 cells. Aspirate and discard the old complete medium. Add 200 μL of PBS and wash the cells once or twice. Add 1 mL of 0.25% Trypsin-EDTA to cover the cell layer and incubate at 37 °C with 5% CO₂ for 3 minutes. Add 3 mL of complete medium to neutralize the trypsin. Gently pipette to resuspend the cells, then transfer them to a centrifuge tube and centrifuge at 200×g for 5 minutes for later use. After centrifugation, discard the supernatant and add 30 μL of PBS. Pipette to resuspend the cells and prepare a single-cell suspension. Add 1 μL of Pacific Blue dye, and incubate at room temperature for 30 minutes. Resuspend in FACS buffer (2% FBS in DPBS), centrifuge at 200×g for 5 minutes, discard the supernatant and wash three times with PBS. Transfer the cells to a 96-well plate, replace with fresh complete medium and incubate at 37 °C with 5% CO₂ for 6 hours until the cells gently adhere to the plate. Add the *S. aureus* stained with carboxyfluorescein succinimidyl ester (CFSE) and incubate with SKOV3 at 37° C for 2 h. 100 μM biotin-hydrazide were added separately to cell mixtures of *S. aureus* tethered to Ru(bpy)_3_^2+^ and SKOV3 cells. After light-induced labeling reaction, wash three times with FACS buffer. Following SA-APC staining, wash three more times with FACS buffer for high-content imaging. Turn on the imaging function of the high-content imaging system and adjust to the 20× objective lens. Observe and capture the morphology and fluorescence expression of the bacteria and cells.

## 14. Flow cytometry and cell sorting

Flow cytometry data analyses were performed using FlowJo software (10.6.2). Cells were suspended in DMEM medium supplemented with APC-streptavidin. First, HeLa cells excluded through cell tracker, Biotin+ HeLa cells excluded through APC-streptavidin. The background was defined as the signal produced on HeLa cells by incubating with *S. aureus* without membrane-anchored Ru(bpy)_3_^2+^.

## 15. RNA-seq analysis

For RNA sequencing (RNA-seq), total RNA was isolated from tumor tissues and measured using a NanoDrop One Microvolume UV-Vis Spectrophotometer (Thermo Fisher Scientific). RNA sample quality was assessed using agarose gel electrophoresis and the Agilent 2100 Bioanalyzer (Agilent Technologies). All samples displayed a 260/280 ratio of around 2.0. Majorbio Biotech constructed and sequenced the cDNA libraries. Briefly, 200 ng of RNA from each group was used for library construction with the TruSeq RNA Sample Prep Kit (Illumina). The constructed DNA was enriched by PCR amplification, then purified using Certified Low Range Ultra Agarose (Bio-Rad) gel electrophoresis. Clone clusters were generated on an IlluminacBot using a Truseq PE Cluster Kit v3-cBot-HS. High-throughput sequencing was then performed on an Illumina MiSeq sequencer using a Truseq SBS Kit v3-HS (200 cycles).

## 16. Data evaluation

The software package limma was used to detect differentially expressed genes. We applied a threshold of *P* < 0.05 and a log fold change of >1.0 for up-regulated genes and <−1.0 for down-regulated genes. Volcano plots and heatmaps, GO analysis and Kyoto Encyclopedia of Genes and Genomes (KEGG) pathway analysis were performed using the majorbio.com. online analysis platform. The Gene Ontology (GO) term analysis and Kyoto Encyclopedia of Genes and Genomes pathway analysis were performed using cluster Profiler.

## 17. RNA extraction and quantitative RT-qPCR

Total RNA was extracted via TRIzol reagent (Ambion 15596-018). Total RNAs (0.5-1 μg) were subjected to reverse transcription with PrimeScript RT Master Mix (Takara, DRR036A). To determine relative mRNA level, RT-qPCR was performed using universal SYBR Green Supermix (Bio-Rad 172-5125) and gene expression was normalized to that of β-actin. Primers used for RT-qPCR were listed in Table S6.

## 18. Bacterial invasion into cells assay

*S. aureus* was stained with CFSE, while HeLa cells were stained with PB 450 dye. After a 4-hour incubation period, differential centrifugation was performed at 100 g for 10 minutes to separate non-invading bacteria from cells adhered to and infected by bacteria. The cells were then washed three times with FACS buffer at 450 g for 5 minutes each, followed by flow cytometric detection of CFSE fluorescence on the cells.

## 19. Statistical analysis

Statistical analyses were performed using GraphPad Prism software (version 9.0). Comparisons between groups were analyzed by two-way ANOVA, and comparisons of multiple samples within a group were analyzed by one-way ANOVA. In all figures with error bars, data are presented as the mean ± SD. In all figures, ns, P > 0.05; *P < 0.05; **P < 0.01; ***P < 0.001; ****P < 0.0001.

## 20. ^1^H NMR, ^13^C NMR, ^31^P NMR and HRMS spectra of compounds

^1^H NMR of compound **3** (600 MHz, D_2_O)

^1^H NMR of compound **4** (600 MHz, D_2_O)

# Supplementary Figures

Figure S1. Characterization of bacteria tethered with Ru(bpy)_3_^2+^ and the activity of Ru(bpy)_3_^2+^ loaded onto bacteria. **(A-C)** Flow cytometry gating strategy and summary analysis of the ratio/median/mean of PerCP-Cy5.5, which represents the amount of Ru(bpy)_3_^2+^ anchored on *E. coli*, *S. aureus* and *B. subtilis* using different concentrations of NHS-[Ru(bpy)₃²⁺]2PF_6_^-^ (0 μM, 0.001 μM, 0.01 μM, 0.1 μM, 1 μM, 10 μM, 100 μM.) **(D-F)** Flow cytometry gating strategy and summary analysis of the ratio/median/mean of self-biotinylation on *E. coli*, *S. aureus* and *B. subtilis* using different concentrations of NHS-[Ru(bpy)₃²⁺]2PF_6_^-^. The bacteria were incubated with NHS-[Ru(bpy)₃²⁺]2PF_6_^-^ for 20 min at room temperature and then irradiated with a 450 nm LED for 5 min. The background is defined as the signal produced on *E. coli*, *S. aureus or* *B. subtilis* without surface-anchored Ru(bpy)_3_^2+^. (n=3, n: number of biological replicates. ns p>0.05; *p<0.05; **p<0.01; ***p<0.001; ****p<0.0001.)

Figure S2. Verfication of the Ru(bpy)_3_^2+^ loading via covalent bonding rather than adhering to the bacteria surface. **(A-C)** Flow cytometry gating strategy and summary analysis of the the ratio/median/mean of PerCP-Cy5.5 representing the amount of Ru(bpy)_3_^2+^, using NHS-[Ru(bpy)₃²⁺]2PF_6_^-^ and DPBS pre-treated NHS-[Ru(bpy)₃²⁺]2PF_6_^-^ (in which the NHS ester was inactivated) for incubation with *E. coli*, *S. aureus* or *B. subtilis*. (n=3, n: number of biological replicates. ns p>0.05; ****p<0.0001.)

Figure S3. Exploration of the effect of loading Ru(bpy)_3_^2+^ onto bacteria and its impact on their viability. **(A-D)** Bacterial growth plates and summary statistics showing bacterial growth 24 h after loading Ru(bpy)_3_^2+^ on *E. coli* and *B. subtilis* by incubation with 0 μM, 0.001 μM, 0.01 μM, 0.1 μM, 1 μM, 10 μM and 100 μM NHS-[Ru(bpy)₃²⁺]2PF_6_^-^*.* (n=3, n: number of biological replicates. ns p>0.05.)

Figure S4. Verification of singlet oxygen mediated labeling reactions. **(A)** Schematic representation of the principle of GSH quenching of singlet oxygen and its application in the self-labeling reaction of *S. aureus* loaded with Ru(bpy)_3_^2+^. **(B, C)** Flow cytometry analysis and summary statistics showing the amount of Ru(bpy)_3_^2+^ loaded on *S. aureus* and the biotinylation ratio on HeLa cells under the treatment of different concentrations of GSH (0 μM; 1 μM; 10 μM; 100 μM). **(D)** Schematic representation of GSH quenching of singlet oxygen and its application in transcellular labeling of *S. aureus* loaded with Ru(bpy)_3_^2+^. **(E, F)** Flow cytometry histogram analysis showing the amount of Ru(bpy)_3_^2+^ loaded on *S. aureus* under the treatment of different concentrations of GSH. **(G, H)** Flow cytometry analysis and summary statistics showing the biotinylation intensity and ratio on HeLa cells under the treatment of different concentrations of GSH (0 μM; 1 μM; 10 μM; 100 μM). The background is defined as the signal produced on HeLa cells when incubated with *S. aureus* without surface-anchored Ru(bpy)_3_^2+^. Incubation time, 2 h; irradiation time, 5 min. n=3. (n: number of biological replicates. ns p>0.05; ***p<0.001; ****p<0.0001.)

Figure S5. Verification of labeling induced by proximity effects between bacteria and host cancer cells. **(A)** Workflow for analyzing the interaction strength of between *S. aureus* and HeLa cells at different ratios (10:1; 5:1; 1:1; 0.5:1) via the “Ru-^1^O_2_-hydrazide” system. **(B, C)** Flow cytometry analysis and summary statistics showing the biotinylation intensity or ratio on HeLa at different ratios of *S. aureus* and HeLa. Incubation time, 2 h; irradiation time, 5 min. n=3. **(D)** Workflow for analyzing the interaction strength between *S. aureus* and HeLa cells at the ratio of 10:1, with the two populations physically separated by a transwell membrane to prevent direct contact via the “Ru-^1^O_2_-hydrazide” system. **(E)** Flow cytometry analysis and summary statistics showing the amount of Ru(bpy)_3_^2+^ loaded on *S. aureus* and the biotinylation intensity or ratio on HeLa cells. The background is defined as the signal produced on HeLa cells when incubated with *S. aureus* without surface-anchored Ru(bpy)_3_^2+^. **(F)** High-content imaging of SKOV3-Ru(bpy)_3_^2+^ after labeling via via the “Ru-^1^O_2_-hydrazide” system, with or without interaction with *S. aureus*. Scale bar: 100 μm. Incubation time, 2 h; irradiation time, 5 min. n=3. (n: number of biological replicates. **p<0.01; ****p<0.0001.)

Figure S6. Quantitative exploration of the “Ru-^1^O_2_-hydrazide” system for studying the interaction between *S. aureus* and HeLa cells. **(A)** Schematic illustration of the quantitative study of the interaction between *S. aureus* and HeLa. **(B, C)** Flow cytometry histograms and summary statistics of the blocking effects of different concentrations of anti-human CD29 antibody on HeLa cells. n=3. **(D, E)** Flow cytometry and summary statistics of the biotinylation ratio on HeLa cells pretreated with anti-human CD29 antibody at concentrations ranging from 0.01 μg/mL to 1 μg/mL (0.01, 0.05, 0.07, 0.5, 0.75 and 1 μg/mL), followed by incubation with *S. aureus-Ru(bpy)_3_^2+^* for 2 hours. The background is defined as the signal produced on HeLa when incubated with *S. aureus* without surface-anchored Ru(bpy)_3_^2+^. Incubation time, 2 h; irradiation time, 5 min. n=3. **(F)** Correlation analysis between the biotinylation ratio on HeLa cells (pretreated with different concentrations of anti-human CD29) and the median fluorescence intensity (MFI) of PE-anti human CD29 following incubation with *S. aureus*-*Ru(bpy)_3_^2+^*. (n: number of biological replicates. ns p>0.05; *p<0.05; ***p<0.001; ****p<0.0001.)

Figure S7. Quantitative exploration of the “Ru-^1^O_2_-hydrazide” system for studying the interaction between *S. aureus* and HeLa cells. **(A)** Schematic illustration of a model of S. aureus adhesion to HeLa cells and the capture of this adhesion process using the “Ru-^1^O_2_-hydrazide” system. **(B, D)** Flow cytometry and summary statistics of the adhesion intensity (median fluorescence intensity of FITC) of *S. aureus* to HeLa cells under different co-incubation times (1, 2, 4 and 6 h). n=3. **(C, E)** Flow cytometry and summary statistics of the biotinylation ratio on HeLa cells via the “Ru-^1^O_2_-hydrazide” system under different co-incubation times (1, 2, 4 and 6 h). The background is defined as the signal produced on HeLa when incubated with *S. aureus* without surface-anchored Ru(bpy)_3_^2+^. Irradiation time, 5 min. n=3. **(F)** Correlation analysis between the biotinylation ratio on HeLa cells and the adhesion intensity (median fluorescence intensity (MFI )of FITC) of *S. aureus* under different co-incubation times (1, 2, 4 and 6 h). **(J, I)** Flow cytometry and summary statistics of the adhesion intensity (median fluorescence intensity of FITC) of *S. aureus* to HeLa cells at different *S. aureus-*to-HeLa ratios (10: 1, 5: 1, 1: 1 and 0.5: 1). n=3. **(H, J)** Flow cytometry and summary statistics of the biotinylation ratio on HeLa cells via the “Ru-^1^O_2_-hydrazide” system at different *S. aureus-*to-HeLa ratios (10: 1, 5: 1, 1: 1 and 0.5: 1). The background is defined as the signal produced on HeLa when incubated with *S. aureus* without surface-anchored Ru(bpy)_3_^2+^. Irradiation time, 5 min. n=3. **(K)** Correlation analysis between the biotinylation ratio on HeLa cells and the adhesion intensity of *S. aureus* (median fluorescence intensity (MFI) of FITC) at different *S. aureus-*to-HeLa ratios (10: 1, 5: 1, 1: 1 and 0.5: 1). (n: number of biological replicates. ns p>0.05; **p<0.01; ****p<0.0001.)

Figure S8. Exploration of the selectivity of the “Ru-^1^O_2_-hydrazide” system for studying the interaction between *S. aureus* and SKOV3 or HeLa cells. **(A, B)** Flow cytometry gating strategy and summary analysis of the ratio of the interaction-dependent biotinylation on SKOV3 cells and the ratio/median/mean of self-anchoring and self-biotinylation on *S. aureus* in the bacteria-cell mixtures when the incubation ratio of *S.aureus*-*Ru(bpy)_3_^2+^* and SKOV3 cells was 10: 1. **(C, D)** Flow cytometry gating strategy and summary analysis of the ratio of the interaction-dependent biotinylation on HeLa cells and the ratio/median/mean of self-anchoring and self-biotinylation on *S. aureus* in the bacteria-cell mixtures when the incubation ratio of *S. aureus*-*Ru(bpy)_3_^2+^* and HeLa cells was 10: 1. The background is defined as the signal produced on when incubated with *S. aureus* without surface-anchored Ru(bpy)_3_^2+^. Incubation time, 2 h; irradiation time, 5 min. (n=3, n: number of biological replicates. **p<0.01; ****p<0.0001.)

Figure S9. Exploring of selectivity of the “Ru-^1^O_2_-hydrazide” system for studying the interaction between *S. aureus* and Jurkat. **(A, B)** Flow cytometric gating strategy and summary analysis of the ratio of the interaction-dependent biotinylation on Jurkat and the ratio/median/mean of self-anchoring and self-biotinylation on *S. aureus* in the bacteria and cell mixtures when the incubation ratio of *S. aureus*-*Ru(bpy)_3_^2+^* and Jurkat was 10: 1. The background is defined as the signal produced on Jurkat when incubating with *S. aureus* without surface-anchored Ru(bpy)_3_^2+^. Incubation time, 2 h; irradiation time, 5 min. (n=3, n: number of biological replicates. ****p<0.0001.)

Figure S10. Exploration of selectivity of the “Ru-^1^O_2_-hydrazide” system for studying the interaction between *S. aureus* and cell mixtures. **(A, B)** Flow cytometry gating strategy and summary analysis of biotinylation on SKOV3 and HeLa cells when *S. aureus*-*Ru(bpy)_3_^2+^* was incubated with a mixture of SKOV3 and HeLa cells (1:1 ratio) at an *S. aureus*-to-cell mixture ratio of 10:1. The background is defined as the signal produced on SKOV3 and HeLa cells when incubated with *S. aureus* without surface-anchored Ru(bpy)_3_^2+^. Incubation time, 2 h; irradiation time, 5 min. (n=3, n: number of biological replicates. ns p>0.05; ****p<0.0001.)

Figure S11. Exploration of selectivity of the “Ru-^1^O_2_-hydrazide” system for studying the interaction between *S. aureus* and cell mixtures. **(A, B)** Flow cytometry gating strategy and summary analysis of biotinylation on HeLa and Jurkat cells when *S. aureus*-*Ru(bpy)_3_^2+^* incubated with a mixture of HeLa and Jurkat cells (1:1 ratio) at an *S. aureus*-to-cell mixture ratio of 10:1. The background is defined as the signal produced on HeLa and Jurkat cells when incubated with *S. aureus* without surface-anchored Ru(bpy)_3_^2+^. Incubation time, 2 h; irradiation time, 5 min. (n=3, n: number of biological replicates. ***p<0.001; ****p<0.0001.)

Figure S12. Use of the “Ru-^1^O_2_-hydrazide” system to study *S. aureus* and primary cells from murine spleen. **(A)** Schematic representation of *S. aureus*-intercellular proximity labeling catalyzed by Ru(bpy)_3_^2+^ in the *S. aureus-*splenocytes interaction. **(B)** Flow cytometry strategy for analyzing the interaction between *S. aureus* and murine splenocytes. **(C-D)** Flow cytometry histogram analysis and summary statistics showing the biotinylation ratio and median or mean of the biotinylation of T cells, B cells and other cells (neither T nor B cells) when *S. aureus* or *S. aureus*-*Ru(bpy)_3_^2+^* interacts with murine splenocytes, n=3. The background is defined as the signal produced on T cell, B cells and other cells (neither T nor B cells) when incubated with *S. aureus* without surface-anchored Ru(bpy)_3_^2+^. Incubation time, 2 h; irradiation time, 5 min. (n: number of biological replicates. **p<0.01; ***p<0.001; ****p<0.0001.)

Figure S13. Use of the *S. aureus-Ru(bpy)_3_^2+^* probe to identify *S. aureus*-HeLa interaction and analyze the underlying interaction mechanisms. **(A)** Expression levels of genes related with malignancy between Biotin+ and Biotin- HeLa cells. n=3. **(B-D)** DO, KEGG and GO terms enriched by the genes related with immune system between Biotin+ and Biotin- HeLa cells. n=3. (n: number of biological replicates. *p<0.05; **p<0.01; ***p<0.001; ****p<0.0001.)

Figure S14. Effects of different metal ions on bacterial and cell viability. **(A, B)** Bacterial growth plates and summary statistics showing *S. aureus* growth 24 h after incubation with different concentrations of 10 μM CuCl_2_, FeCl_3_, FeCl_2_, MnCl_2_, AlCl_3_, KCl, LiCl, CaCl_2_, NaCl, CoCl_2_ and NiCl_2_ respectively. **(C)** Summary statistics showing the HeLa cell viability after treatment with different concentrations of metal ions. (0 μM, 10 μM, 50 μM, 100 μM, 200 μM) (n: number of biological replicates. ns p>0.05; *p<0.05; **p<0.01.)

Figure S15. Use of *S. aureus-Ru(bpy)_3_^2+^* probe to identify the effects of different metal ions on *S. aureus*-HeLa interaction. **(A)** Flow cytometry gating strategy for analyzing the ratio of interaction-dependent biotinylation on HeLa cells when incubated with 10 μM Cu^2+^, Fe^3+^, Fe^2+^, Mn^2+^, Mg^2+^, Al^3+^, Li^+^, Ca^2+^, Na^+^, Co^2+^ and Ni^2+^. The ratio of *S. aureus*-*Ru(bpy)_3_^2+^* and HeLa cells was 10: 1. The background is defined as the signal produced on HeLa cells when incubated with *S. aureus* without surface-anchored Ru(bpy)_3_^2+^ and with no metal ions treatment. Metal ions treatment time, 4 h; Incubation time, 2 h; irradiation time, 5 min. (n=3, n: number of biological replicates. ns p>0.05; *p<0.05; **p<0.01; ****p<0.0001.)

Figure S16. Use of *S. aureus-Ru(bpy)_3_^2+^* probe to identify the effects of different metal ions on *S. aureus*-HeLa interaction. **(A)** Flow cytometric gating strategy and summary of the ratio of the interaction-dependent biotinylation on HeLa cells when incubated with 10 μM Cu^2+^, Fe^3+^, Fe^2+^, Mn^2+^, Mg^2+^, Al^3+^, Li^+^, Ca^2+^, Na^+^, Co^2+^ and Ni^2+^. The ratio of *S. aureus*-*Ru(bpy)_3_^2+^* and HeLa was 10: 1. The background is defined as the signal produced on HeLa cells when incubated with *S. aureus* without surface-anchored Ru(bpy)_3_^2+^ and with no metal ions treatment. Metal ions treatment time, 4 h; Incubation time, 2 h; irradiation time, 5 min. (n=3, n: number of biological replicates. ns p>0.05; *p<0.05; **p<0.01; ***p<0.001; ****p<0.0001.)

Figure S17. Evaluation of metal ions in modulating bacteria-host cell interactions via the “Ru-^1^O_2_-hydrazide” system. **(A-D)** Reactome, GO, DO and KEGG enrichment analysis of the DEGs between Biotin+ (Mn^2+^ treated) and Biotin+ (untreated) HeLa cells. n=3.

Figure S18. Analysis of the effects of Mn^2+^ on *S. aureus*-HeLa interactions. **(A-D)** GSEA of ubiquitinyl hydrolase, thiol-dependent ubiquitinyl hydrolase activity, thiol-dependent ubiquitinyl-specific protease activity and protein sumoylation in Mn^2+^ treated Biotin+ HeLa cells, P<0.001. n=3. **(E-H)** Heatmap generated from GSEA using the GO database.

Figure S19. Analysis of the effects of Mn^2+^ on *S. aureus*-HeLa interactions. (A) Bar chat showing the expression levels of the genes associated with cancer cell malignancy in Mn^2+^ treated Biotin+ HeLa cells compared to untreated Biotin+ HeLa cells. n=3. (n: number of biological replicates. **p<0.01; ***p<0.001; ****p<0.0001.)

# Supplementary Tables

| **Gene ID** | **Gene Name** | **Gene Function** |
| --- | --- | --- |
| ENSG00000258017 | *TUBA1B-AS1* | As a prognostic and immunobiological marker of cancer in both pancreatic and breast cancers, and has been shown to mediate cancer cell growth^45,46^. |
| ENSG00000175063 | *UBE2C* | Encoding a key enzyme that initiates the labeling process which triggers cell division, thereby acting as a central regulator of mitosis^47^. |
| ENSG00000170312 | *CDK1* | Serves crucial regulator of cell mitosis; complexes with Cyclin B and acts as a downstream substrate for serine/threonine protein kinase phosphorylation^48^. |
| ENSG00000146674 | *IGFBP3* | Encoding a protein that acts as a molecular dispatcher that regulates the expression of the growth factor IGF, thereby controlling cell growth or death^49^. |
| ENSG00000096433 | *ITPR3* | A downregulated gene; mediate calcium ion release from the endoplasmic reticulum^50^. |
| ENSG00000089154 | *GCN1* | Regulation of the initiation of cellular stress responses^51^. |
| ENSG00000197102 | *DYNC1H1* | Controls the dynein motor complex, the complex is responsible for retrograde transport in the cell centre^52^. |
| ENSG00000125730 | *C3* | Regulation of tumor metastasis^53^. |
| ENSG00000008710 | *PKD1* | Regulation of key target proteins associated with cancer therapy^54^. |
| ENSG00000176986 | *SEC24C* | Downregulation of this gene enhances T-cell activation and anti-tumor immunity^55^. |

## Table S1. Functional list of genes highlighted in the volcano plot comparing Biotin+ and Biotin- HeLa cells.

**List of all differentially genes (DEGs) related to cell membrane proteins between Biotin+ HeLa and Biotin- HeLa cells**

| **I. Cell Cycle and Proliferation** | | | | | | |
| --- | --- | --- | --- | --- | --- | --- |
| Gene ID | Gene names | Gene description | FC(LC/control) | P value | Significant | Regulate |
| ENSG00000170312 | *CDK1* | Cell cycle control (G2/M transition, mitosis) | 2.903228169 | 1.08E-154 | Yes | up |
| ENSG00000092036 | *HAUS4* | Microtubule spindle assembly (HAUS complex) | 0.464637678 | 2.05E-37 | Yes | down |
| ENSG00000166106 | *ADAMTS15* | Extracellular matrix proteolysis, angiogenesis | 0.364931098 | 3.25E-09 | Yes | down |
| **II. Signal Transduction and Cellular Communication** | | | | | | |
| ENSG00000166428 | *PLD4* | Phospholipid metabolism, immune regulation | 4.767483188 | 7.21E-20 | Yes | up |
| ENSG00000125910 | *S1PR4* | Sphingosine-1-phosphate receptor, immune cell migration | 2.446134423 | 4.72E-08 | Yes | up |
| ENSG00000231738 | *TSPAN19* | Tetraspanin family, cell membrane organization | 2.234960545 | 0.000935472 | Yes | up |
| ENSG00000258644 | *SYNJ2BP-COX16* | RNA gene (function unclear), potentially mitochondrial | 9.544008687 | 0.002505527 | Yes | up |
| ENSG00000096433 | *ITPR3* | Calcium release from endoplasmic reticulum | 0.43630929 | 9.6E-155 | Yes | down |
| ENSG00000124181 | *PLCG1* | Phospholipase signaling, calcium pathway activation | 0.470781354 | 2.36E-41 | Yes | down |
| ENSG00000171608 | *PIK3CD* | Phosphoinositide 3-kinase, cell growth signaling | 0.46829067 | 1.94E-08 | Yes | down |
| ENSG00000248592 | *STIMATE-MUSTN1* | STIM modulator (calcium signaling), muscle development | 0.466316089 | 0.000763649 | Yes | down |
| **III. Substance Transport and Metabolism** | | | | | | |
| ENSG00000227039 | *ITGB2-AS1* | ITGB2-AS1 is a long non-coding RNA that primarily acts as an oncogene. | 2.846487936 | 0.0000455 | Yes | up |
| ENSG00000176986 | *SEC24C* | COPII vesicle cargo sorting (ER export) | 0.479165355 | 3.69E-69 | Yes | down |
| ENSG00000292982 | *WASH5P* | Non-coding RNA (function unclear, pseudogene) | 0.402225421 | 1.1E-37 | Yes | down |
| ENSG00000138031 | *ADCY3* | cAMP synthesis, G-protein signaling | 0.491043322 | 2.71E-37 | Yes | down |
| ENSG00000124574 | *ABCC10* | Multidrug resistance transporter | 0.467699364 | 2.78E-15 | Yes | down |
| ENSG00000103061 | *SLC7A6OS* | Amino acid transporter subunit (y+LAT complex) | 0.483237763 | 2.24E-13 | Yes | down |
| ENSG00000171017 | *LRRC8E* | Volume-regulated anion channel (VRAC) subunit | 0.484163859 | 6.23E-12 | Yes | down |

## Table S2. List of all differentially expressed genes (DEGs) encoding cell membrane proteins in Biotin+ versus Biotin- HeLa cells.

**List of all differentially genes (DEGs) related to cell membrane proteins between Biotin+ HeLa with Mn^2+^ treatment and Biotin+ HeLa cells with no treatment**

| **I. Signal Transduction and Cellular Communication** | | | | | | |
| --- | --- | --- | --- | --- | --- | --- |
| Gene ID | Gene names | Gene description | FC(LC/control) | P value | Significant | Regulate |
| ENSG00000117335 | *CD46* | Complement regulator/pathogen receptor | 2.236080975 | 3.90E-283 | Yes | up |
| ENSG00000179295 | *PTPN11* | Encodes SHP-2 phosphatase, regulates cell signaling (e.g., RAS/MAPK). | 2.551451779 | 2.44E-103 | Yes | up |
| ENSG00000133657 | *ATP13A3* | Polyamine transporter, important for cardiovascular development. | 2.586932187 | 7.54E-80 | Yes | up |
| ENSG00000068650 | *ATP11A* | Lipid flippase; maintains plasma membrane asymmetry. | 2.543051402 | 5.68E-58 | Yes | up |
| ENSG00000152894 | *PTPRK* | Receptor phosphatase; involved in cell adhesion. | 2.556354522 | 2.75E-30 | Yes | up |
| ENSG00000154639 | *CXADR* | Primary receptor for coxsackievirus and adenovirus. | 2.009565824 | 3.29E-28 | Yes | up |
| ENSG00000125257 | *ABCC4* | Drug transporter (MRP4); effluxes nucleotides and drugs. | 2.441002488 | 1.88E-27 | Yes | up |
| ENSG00000085563 | *ABCB1* | Drug transporter (P-gp); confers multidrug resistance. | 3.555766571 | 1.25E-18 | Yes | up |
| ENSG00000101974 | *ATP11C* | Lipid flippase for phosphatidylserine; important for B-cells. | 2.008595473 | 4.63E-14 | Yes | up |
| ENSG00000144724 | *PTPRG* | Tumor suppressor phosphatase; regulates cell growth. | 3.601998401 | 0.000000315 | Yes | up |
| ENSG00000198821 | *CD247* | Signaling subunit of the T-cell receptor (TCR). | 5.3032464 | 0.00000271 | Yes | up |
| ENSG00000118777 | *ABCG2* | Drug transporter (BCRP); protects cells from toxins. | 2.398634691 | 0.00000768 | Yes | up |
| ENSG00000261286 | *ATP2C2-AS1* | Long non-coding RNA; function under investigation. | 2.497443566 | 0.020424473 | Yes | up |
| ENSG00000167775 | *CD320* | Receptor for vitamin B12 (cobalamin) uptake. | 0.284787754 | 6.47E-185 | Yes | down |
| ENSG00000129226 | *CD68* | Scavenger receptor; marker for macrophages and monocytes. | 0.135325865 | 7.55E-175 | Yes | down |
| ENSG00000173264 | *GPR137* | Orphan G-protein coupled receptor; function not fully characterized. | 0.360421814 | 5.11E-57 | Yes | down |
| ENSG00000170412 | *GPRC5C* | Retinoic acid-inducible GPCR; role in cell adhesion and differentiation. | 0.363916275 | 4.32E-43 | Yes | down |
| ENSG00000160683 | *CXCR5* | Chemokine receptor for CXCL13; guides B-cells to lymphoid follicles. | 0.000138663 | 2.05E-27 | Yes | down |
| ENSG00000178623 | *GPR35* | GPCR activated by kynurenic acid; involved in immune and metabolic processes. | 0.300784424 | 6.04E-27 | Yes | down |
| ENSG00000080031 | *PTPRH* | Receptor-type phosphatase; putative tumor suppressor. | 0.411320726 | 3.7E-15 | Yes | down |
| ENSG00000181773 | *GPR3* | GPCR that constitutively activates signaling; involved in maintaining meiotic arrest. | 0.368279054 | 6.3E-13 | Yes | down |
| ENSG00000158292 | *GPR153* | Orphan GPCR; predicted role in neurotransmission. | 0.438955768 | 8.56E-13 | Yes | down |
| ENSG00000105204 | *DYRK1B* | Serine/threonine kinase; regulates insulin signaling and cell proliferation. | 0.275807116 | 0.001232033 | Yes | down |
| ENSG00000125726 | *CD70* | Ligand for CD27; costimulatory signal for T-cell and B-cell activation. | 0.179703155 | 0.023102051 | Yes | down |
| **II: Cell Adhesion and Connections** | | | | | | |
| ENSG00000177697 | *CD151* | Tetraspanin protein; regulates cell adhesion, migration and signal transduction. | 0.352246977 | 1.31E-219 | Yes | down |
| ENSG00000189143 | *CLDN4* | Claudin 4; component of tight junctions, regulates paracellular permeability. | 0.321704925 | 1.68E-121 | Yes | down |
| ENSG00000181885 | *CLDN7* | Claudin 7; component of tight junctions, crucial for epithelial integrity. | 0.406114583 | 1.09E-115 | Yes | down |
| ENSG00000002586 | *CD99* | Involved in leukocyte migration, T-cell adhesion and differentiation. | 0.487584649 | 1.37E-30 | Yes | down |
| ENSG00000085117 | *CD82* | Tetraspanin protein; metastasis suppressor, regulates cell motility and signaling. | 0.487691873 | 2.37E-10 | Yes | down |
| ENSG00000213937 | *CLDN9* | Claudin 9; component of tight junctions, important in ion selectivity. | 0.290258272 | 0.0000594 | Yes | down |
| **III: Vesicular Trafficking and Membrane** | | | | | | |
| ENSG00000132842 | *AP3B1* | Adaptor protein complex subunit (vesicle formation) | 2.067333808 | 2.03E-55 | Yes | up |
| ENSG00000119541 | *VPS4B* | ESCRT complex ATPase (membrane scission) | 2.727388593 | 3.52E-38 | Yes | up |
| ENSG00000144036 | *EXOC6B* | Exocyst complex component (vesicle tethering) | 2.334363519 | 4.76E-36 | Yes | up |
| ENSG00000070367 | *EXOC5* | Exocyst complex component (vesicle docking) | 2.07274167 | 7.73E-30 | Yes | up |
| ENSG00000129003 | *VPS13C* | Lipid transfer protein (membrane contact sites) | 2.738593754 | 1.453435271 | Yes | up |
| ENSG00000116903 | *EXOC8* | Exocyst complex component (vesicle transport) | 2.168081763 | 5.92E-28 | Yes | up |
| ENSG00000112685 | *EXOC2* | Exocyst complex component (vesicle tethering) | 2.279722391 | 3.59E-24 | Yes | up |
| ENSG00000041353 | *RAB27B* | Small GTPase (secretory vesicle regulation) | 2.8064272 | 7.83E-23 | Yes | up |
| ENSG00000143952 | *VPS54* | GARP complex subunit (retrograde transport) | 2.120835157 | 2.33E-20 | Yes | up |
| ENSG00000138190 | *EXOC6* | Exocyst complex component | 2.144183727 | 1.07E-14 | Yes | up |
| ENSG00000104915 | *STX10* | Syntaxin, regulates the fusion of neurotransmitter vesicles with the presynaptic membrane. | 0.337098719 | 3.38E-116 | Yes | down |
| ENSG00000105649 | *RAB3A* | Rab GTPase, regulates exocytosis-endosome fusion | 0.321143598 | 2.37E-20 | Yes | down |
| ENSG00000106089 | *STX1A* | Rab GTPase, regulates the docking and fusion of neurotransmitter/hormone vesicles. | 0.349165683 | 1.28E-16 | Yes | down |
| ENSG00000179044 | *EXOC3L1* | Exocyst complex-associated protein, regulates epithelial secretion and tumor metastasis. | 0.140817362 | 0.00465496 | Yes | down |

## Table S3. List of differentially expressed genes (DEGs) encoding cell membrane proteins in Mn^2+^ treated and untreated Biotin+ HeLa cells.

| Gene ID | Gene Name | Gene Function |
| --- | --- | --- |
| ENSG00000008517 | *IL32* | Proinflammation and procancer. Creating an inflammatory environment in the tumor microenvironment activates pathways such as NF-κB, which is associated with the progression of pancreatic cancer, lung cancer, and other cancers^79^. |
| ENSG00000034510 | *TMSB10* | They are highly expressed in liver cancer and colorectal cancer, and promote proliferation, migration and inhibit apoptosis by regulating the cytoskeleton^80^. |
| ENSG00000042493 | *CAPG* | It remodels the cytoskeleton and enhances the motility of cancer cells^81^. |
| ENSG00000051523 | *CYBA* | As a component of NADPH oxidase, it generates reactive oxygen species (ROS) that act as signaling molecules to promote tumor growth^82^. |
| ENSG00000076944 | *STXBP2* | It is involved in vesicular transport and drives tumor growth by influencing specific signaling pathways in certain cancers^83^. |
| ENSG00000084207 | *GSTP1* | It induces tumor drug resistance by metabolizing and eliminating chemotherapeutic drugs^84^. |
| ENSG00000100097 | *LGALS1* | It is highly expressed in a variety of cancers and drives malignant progression by promoting angiogenesis and inhibiting T-cell function^85^. |
| ENSG00000101210 | *EEF1A2* | It is highly expressed in ovarian cancer, breast cancer, and other cancers. It not only undertakes translational functions but also activates pro-survival pathways such as PI3K/Akt, thereby promoting cancer cell proliferation and metastasis^86^. |
| ENSG00000102854 | *MSLN* | It is highly expressed in mesothelioma, pancreatic cancer, and ovarian cancer, promoting proliferation, migration, and invasion while inhibiting apoptosis. It is a promising target for immunotherapy and targeted therapy^87^. |
| ENSG00000105701 | *FKBP8* | It protects cancer cells from apoptosis by inhibiting the Bcl-2/Bax pathway, and is associated with chemoresistance and poor prognosis in a variety of cancers^88^. |
| ENSG00000106211 | *HSPB1* | As a molecular chaperone, it protects cancer cells from stress-induced death and strongly promotes invasion, metastasis, and treatment resistance^89^. |
| ENSG00000107159 | *CA9* | It is highly expressed in various solid tumors such as renal cell carcinoma, induced by HIF-1α, and regulates pH balance to promote tumor growth, invasion, and metastasis^90^. |
| ENSG00000108106 | *UBE2S* | It drives cell cycle progression by mediating the degradation of target proteins, and its high expression is associated with genomic instability and poor prognosis^91^. |
| ENSG00000110195 | *FOLR1* | It is highly expressed in various epithelial cancers such as ovarian cancer and lung cancer, serves as key target for tumor-targeted therapy, and promotes folate uptake and proliferation of cancer cells^92^. |
| ENSG00000126432 | *PRDX5* | As a peroxidase, it scavenges reactive oxygen species (ROS), protects cancer cells from oxidative stress-induced damage, and promotes their survival, chemoresistance, and metastasis. Its role varies across different types of cancer, but most studies have demonstrated its pro-cancer properties^93^. |
| ENSG00000130303 | *BST2* | It is highly expressed in a variety of cancers and promotes proliferation, invasion, and metastasis by activating signaling pathways such as NF-κB. It is also used as biomarker for certain cancers^94^. |
| ENSG00000130600 | *H19* | It strongly promotes cancer cell proliferation, metastasis, and treatment resistance, and is associated with the properties of cancer stem cells^95^. |
| ENSG00000130821 | *SLC6A8* | It is responsible for transporting creatine into cells, providing rapidly regenerated ATP for cancer cells with high energy demands, and directly supporting their proliferation, invasion and survival^96^. |
| ENSG00000136830 | *NIBAN2* | It significantly enhances the viability, treatment resistance, and metastatic potential of cancer cells  by inhibiting apoptosis, promoting autophagy, and adapting to stressful environments, and is associated with poor prognosis in a variety of cancers^97^. |

## Table S4. Functional list of downregulated genes associated with cancer malignancy by GSEA in Mn^2+^ treated versus untreated Biotin+ HeLa cells.

| **Gene ID** | **Gene Name** | **Gene Function** |
| --- | --- | --- |
| ENSG00000071794 | *HLTF* | It possesses SWI/SNF chromatin remodeling activity and E3 ubiquitin ligase activity, and is involved in DNA damage repair. It is often silenced due to promoter methylation, with a function similar to that of a tumor suppressor gene^98^. |
| ENSG00000080824 | *HSP90AA1* | It is the major isoform of HSP90, responsible for stabilizing hundreds of "client proteins", many of which are key oncoproteins and kinases. Cancer cells are highly dependent on it to maintain protein stability and rapid proliferation, making it a crucial anti-cancer target^99^. |
| ENSG00000109971 | *HSPA8* | It is involved in protein folding, trafficking and autophagy. Highly expressed in cancer cells, it acts by inhibiting apoptosis, promoting autophagy and stabilizing oncoproteins^100^. |
| ENSG00000072501 | *SMC1A* | Core subunit of the cohesin complex. Similar to RAD21, it is a key protein for chromosome segregate ion and DNA repair. Its dysfunction leads to genomic instability, a hallmark of cancer. It is highly expressed in various tumors and associated with proliferation and poor prognosis^101^. |
| ENSG00000263001 | *GTF2I* | Transcription factors and oncogenes. This gene serves as a crucial oncogenic driver, and somatic mutations in it are frequently detected, especially in thymomas^102^. |
| ENSG00000167986 | *DDB1* | DNA Damage Repair and Ubiquitin Ligase Adaptor. It is a core component of the CRL4 ubiquitin ligase complex, involved in nucleotide excision repair to respond to UV damage, and regulates the cell cycle, apoptosis, and metabolism^103^. |
| ENSG00000253729 | *PRKDC* | Catalytic subunit of DNA-PK. It is the core kinase in the non-homologous end joining (NHEJ) path way for DNA double-strand break repair^104^. |
| ENSG00000260032 | *NORAD* | Long non-coding RNA (lncRNA). It is a potent negative regulator of the DNA damage response. Acting as a molecular sponge, it binds to and sequesters PUMILIO proteins, preventing them from degrading pro-survival mRNAsepair^105^. |

## Table S5. Functional list of upregulated genes associated with ubiquitination by GSEA in Mn^2+^ treated versus untreated Biotin+ HeLa cells.

| **Gene name** | **Primer name** | **sequences (5' to 3')** |
| --- | --- | --- |
| **β-actin** | β-actin-F | TCCCTGGAGAAGAGCTACGA |
|  | β-actin-R | AGCACTGTGTTGGCGTACAG |
| **UBE2C** | UBE2C-F | CGAGTTCCTGTCTCTCTGCC |
|  | UBE2C-R | CAGCTCCTGCTGTAGCCTTT |
| **CDK1** | CDK1-F | AAACTACAGGTCAAGTGGTAGCC |
|  | CDK1-R | TCCTGCATAAGCACATCCTGA |
| **IGFBP3** | IGFBP3-F | GCGCCAGGAAATGCTAGTG |
|  | IGFBP3-R | GGAACTTGGGATCAGACACCC |
| **CCN5** | CCN5-F | TGTGCCCGACACCATGTACC |
|  | CCN5-R | CCACAGCCATCCAGCACCAG |
| **EDN2** | EDN2-F | TTGGACATCATCTGGGTGAA |
|  | EDN2-R | CTGTAGTGGCCCCTGTCTTG |
| **DYNC1H1** | DYNC1H1-F | TTGCGGCCCTATGGTGAAAT |
|  | DYNC1H1-R | GGATCATCTGCTCCACCTCG |
| **C3** | C3-F | ATACCAAAAGGACGCCCCTG |
|  | C3-R | CAAGGTGCCTTGGCCTTTTC |
| **SEC24C** | SEC24C-F | GTGTTGAAGAGTGATGTCCTG |
|  | SEC24C-R | GCTGGTGGTTCGGTAGTA |
| **PPP2R5D** | PPP2R5D-F | ATCTACGAGACGGAGCAT |
|  | PPP2R5D-R | TAGGACACGGATGAGGAA |
| **TUBA1B-AS1** | TUBA1B-AS1-F | AGTTGTGGTTCGGATAGC |
|  | TUBA1B-AS1-R | GTTGTCATCAGGCACCTA |

## Table S6. Primer sequences used for q-PCR.

# Author Contributions

S. Q., X. H. Z, and X. L. designed the experimental strategies and wrote the manuscript. A. M. S., K. H. W., H. F. S. and K. M. T. performed the experiments. S. Q. and A. M. S. prepared the figures, analyzed the data and edited the manuscript. All authors contributed to the preparation of the manuscript. S. Q. is the lead contact.

# Reference

1. J. Org. Chem., 1989, 54(7), 1731–1735.
